# Molecular Dynamics Simulation of the Crystallizable Fragment of IgG1—Insights for the Design of Fcabs

**DOI:** 10.3390/ijms15010438

**Published:** 2014-01-02

**Authors:** Balder Lai, Christoph Hasenhindl, Christian Obinger, Chris Oostenbrink

**Affiliations:** 1 Department of Material Sciences and Process Engineering, Institute of Molecular Modeling and Simulation, BOKU (University of Natural Resources and Life Sciences), Vienna A-1190, Austria; E-Mail: balder.lai@boku.ac.at; 2 Department of Chemistry, Division of Biochemistry, VIBT (Vienna Institute of BioTechnology), BOKU (University of Natural Resources and Life Sciences), Vienna A-1190, Austria; E-Mails: christoph.hasenhindl@boku.ac.at (C.H.); christian.obinger@boku.ac.at (C.O.)

**Keywords:** molecular dynamics simulations, molecular modeling, crystallizable Fc fragment, monoclonal antibody

## Abstract

An interesting format in the development of therapeutic monoclonal antibodies uses the crystallizable fragment of IgG1 as starting scaffold. Engineering of its structural loops allows generation of an antigen binding site. However, this might impair the molecule’s conformational stability, which can be overcome by introducing stabilizing point mutations in the CH3 domains. These point mutations often affect the stability and unfolding behavior of both the CH2 and CH3 domains. In order to understand this cross-talk, molecular dynamics simulations of the domains of the Fc fragment of human IgG1 are reported. The structure of human IgG1-Fc obtained from X-ray crystallography is used as a starting point for simulations of the wild-type protein at two different pH values. The stabilizing effect of a single point mutation in the CH3 domain as well as the impact of the hinge region and the glycan tree structure connected to the CH2 domains is investigated. Regions of high local flexibility were identified as potential sites for engineering antigen binding sites. Obtained data are discussed with respect to the available X-ray structure of IgG1-Fc, directed evolution approaches that screen for stability and use of the scaffold IgG1-Fc in the design of antigen binding Fc proteins.

## 1. Introduction

The concept of specifically targeting one molecule using a different molecule was first mentioned in Ehrlich’s side-chain theory which eventually led to the well-known “magic bullet” concept, suggesting compounds that have highly specific targets [1]. Ehrlich’s suggestion [2] that the immune system works in a similar way, where antibodies are the magic bullets, was realized by the development of hybridoma technology for monoclonal antibody (mAb) production by Köhler and Milstein [1,3,4], and by significant advances made in mAb production technologies in the four decades to follow. These advances have allowed the transition from murine mAbs to chimeric mAbs, then to humanized mAbs and finally to therapeutically favorable fully humanized mAbs [4–6]. The first to be described was therapeutically unfavorable due to immunogenicity issues that could be ascribed to different glycosylation in mouse and human. The second and third substantially diminished immunogenicity issues by crafting either the complementarity determining regions (CDRs) which are responsible for antigen recognition and binding, or the whole variable region (Fv) containing the CDRs, onto a human(ized) scaffold. The fourth and most recent example to appear on the market was achieved by using transgenic mice to produce mAbs [7]. Similar advances have also been achieved with different expression systems, such as phage, yeast or plants [8–11]. It is these advances that set the stage for the development of therapeutic mAbs. Initially, targeted diseases were various cancers and infectious diseases as well as some immunological diseases [12]. The number of possible targets for mAbs continues to expand and includes, e.g., the human immunodeficiency virus (HIV) [13], Alzheimer [14] and G-protein coupled receptors [15].

However, even with better understanding of immunogenicity and high efficacy, therapeutic mAbs still rely on mutagenesis or glycoengineering to control antibody-dependent, cell-mediated cytotoxicity (ADCC), structural stability, pharmacokinetics and (pH-dependent) antigen binding [16]. Additionally, it is possible to use completely different formats, e.g., antibody fragments [17], which is mainly the result of a collection of combinatorial approaches to reduce the size of a full-size mAb of the immunoglobulin G class (150 kDa). These smaller fragments include single-chain variable fragments (scFvs; 27 kDa), minibodies (80 kDa), and various scFv- and Fab-based multimers [18,19]. Recently, even smaller alternative binding domains have been engineered (e.g., DARPins or affibodies [20–23]) and, more recently, there has been a rapid increase in design of multifunctional antibodies with multiple binding scaffolds. However, many of these new formats suffer from the absence of binding sites for ligands that trigger ADCC, complementary dependent cytotoxicity (CDC) or mediate a long half-life.

**Figure 1.**
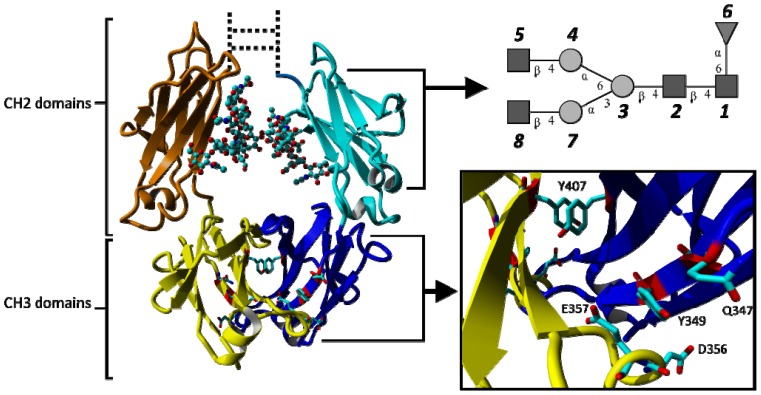
Graphical representation of the crystallizable fragment (Fc) of human IgG1 consisting of two *N*-glycosylated CH2 domains and two CH3 domains (individual domains are shown in different colours). Coordinates were taken from the crystal structure 1OQO. The glycosylation is shown in ball and stick representation and schematically in the top-right panel. Y407 located at the interface of the two CH3 domains forms a favorable stacking interaction. The position of the point mutation Q347 and adjacent residues affected by the exchange of glutamine by glutamate (Q347E) are shown in stick representation. Glycan numbering reflects the order in which they are attached to the topologies used for simulations (GlcNac: *1*, *2*, *5*, *8*; Mannose: *3*, *4*, *7*; Fucose: *6*). The hinge region and disulphide bonds connecting the two CH2 domains are shown as dotted lines.

Among more recent developments in therapeutic mAbs are approaches that focus on the crystallizable fragment (Fc) of immunoglobulin G1 (IgG1), either in its dimeric form as starting scaffold [24] or as monomeric fragments to enhance the half-life of other proteins [25,26]. The Fc protein has—with the exception of an antigen binding site—all the properties of a full-size IgG, *i.e.*, the ability to bind Fcγ-receptors (leading to ADCC), the complementary activator C1q (leading to CDC) and neonatal Fc-binding receptor (improving half-life). Figure 1 shows a graphical representation of human IgG1-Fc (PDB ID 1OQO). The Fc protein consists of two monomers, chain A and B, each comprising an *N*-glycosylated CH2 domain and a CH3 domain. The CH2 domains are *N*-terminally connected by a hinge region that contains two disulfide bridges. Each CH3 domain provides three *C*-terminal (structural) loops that are diversified for the generation of novel antigen binding sites: residues 358–362 (AB-loop), residue 383–391 (CD-loop) and residues 413–422 (EF-loop) (Eu numbering according to Kabat *et al*. [27]). These new formats are called Fcabs, antigen binding Fc proteins, and are of interest as novel therapeutic antibodies by themselves or to further develop bispecific antibodies [28].

However, engineering of structural loops in Fcabs might impair the molecule’s conformational stability. This can be overcome by the introduction of stabilizing point mutations. It has been observed in experiments that such point mutations affect the stability and unfolding behavior of both the CH2 and CH3 domains [29,30]. Some changes to properties of mAbs can be understood from experimental measurements only, but others require a more detailed rationalization at the atomic level. In many of such cases, molecular dynamics (MD) simulations may be used. Examples include MD simulations of mAb-antigen interactions [31,32], analysis of the impact of thermal stress and deglycosylation on the conformational stability of different domains of a murine immunoglobulin G2 (IgG2) mAb [33] or of the role of individual amino acids on the internal dynamics of a full humanized IgG1-based mAb, trastuzumab, or fragments of it [34].

In this study, MD simulations were used to gain a better understanding of the structural stability and dynamics of the CH2 and CH3 domains in IgG1-Fc. In particular, the impact of the hinge region and the *N*-glycosylation at the CH2 domains as well as the effect of two different protonation states (corresponding to pH ≈ 4 and pH ≈ 7) on the structure and dynamics of IgG1-Fc is investigated. Moreover, we consider the effect of an experimentally described point mutation in the CH3 domain (Q347E) [30]. The obtained data are discussed with respect to the X-ray structure(s) of IgG1-Fc proteins and engineering strategies for the design of Fcabs.

## 2. Results and Discussion

### 2.1. Results

Five molecular dynamic simulations of the crystallizable Fc fragment of IgG1 (IgG-Fc) were performed. Two simulations were based directly on the structure with PDB ID 1OQO, one at pH ≈ 4 (B4) and one at pH ≈ 7 (B7). In two additional simulations (H4 and H7), the hinge region connecting the *N*-termini of the CH2 domains were modeled (see methods). Finally, the Q347E mutant was simulated in the presence of the hinge at pH ≈ 7.

#### 2.1.1. Structural Stability and Domain Dynamics

Time series of the atom-positional root-mean-square deviations (RMSD) are shown in Figures S1–S5 (see Supplementary Information). Except for simulation B4 and to some extent H4, the RMSD-values of all domains remain fairly constant after the first 10 ns of simulation, suggesting that an equilibrium state was reached. The RMSD values of CH2 domains typically level off at 0.15 nm during the simulations, while the CH3 domains reach a value between 0.20 and 0.30 nm. The RMSD values of the glycans increase to 0.30 nm. In the simulation of the Fc fragment without hinge region at pH ≈ 4 (simulation B4), the RMSD value of the glycans reaches 0.4 nm and the RMSD value of the CH2 and CH3 domains are larger in comparison to the other four simulations. These findings suggest that the CH2 domains are slightly more stable than the CH3 domains and that in an acidic environment the flexibility of the glycans and the domains is increased. At pH ≈ 4.0, which corresponds roughly to the pH of IgG-Fc elution from Protein A column (*i.e.*, pH ≈ 3.8), the structural integrity of the protein is still intact.

**Figure 2.**
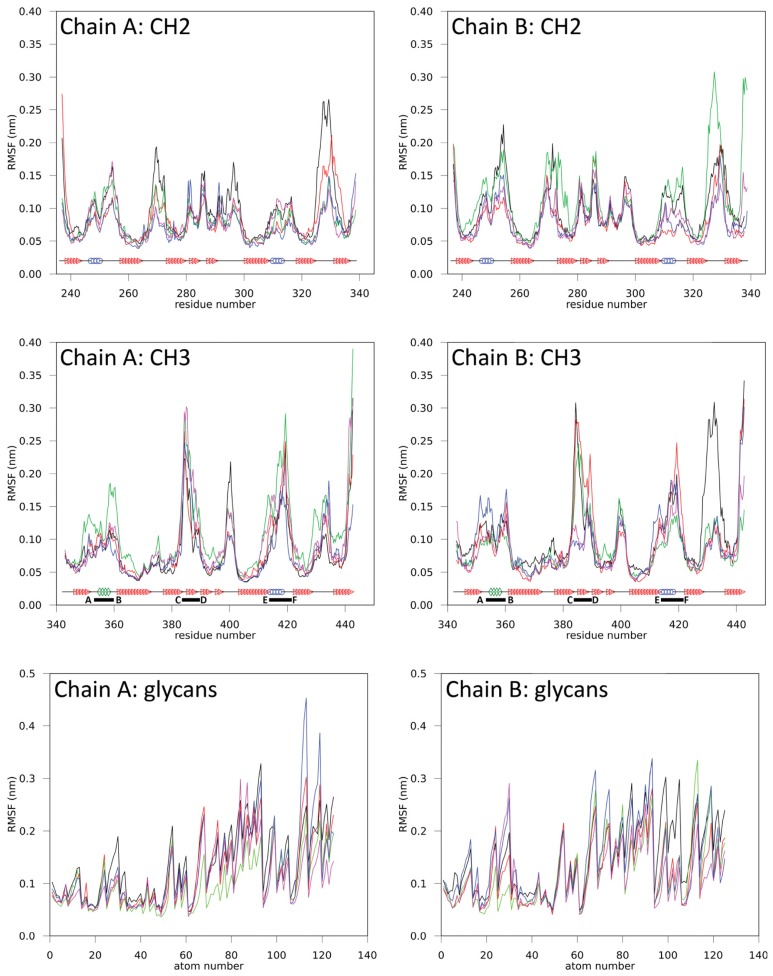
Atom-positional root-mean-square fluctuations (RMSF) for backbone atoms in various domains and glycans in simulations B4 (*black*), B7 (*red*), H4 (*green*), H7 (*blue*) and Q347E (*magenta*). CH2 and CH3 domain secondary structures are shown: helices (*blue circle*), 3/10 helices (*green diamonds*), β-sheets (*red triangle*) and coils (*black line*). *C*-terminal loop regions are indicated by corresponding letters and a solid black bar, which specifies the name and range of the loop.

The atom-positional root-mean-square fluctuations (RMSF) of backbone atoms and glycans are shown in Figure 2. Even if the magnitude of the values differs, the individual domains show a distinctive RMSF fingerprint. Average RMSF values are given in Table S1 (see Supplementary Information). The CH2 domains have characteristic regions of higher RMSF values between residues 246–258, 264–276, 278–301, 307–318 and 321–336 of the respective domain (Figure 2). Increased fluctuation is mainly seen at the structural loops connecting the β-strands (A–G) in the CH2 domains but also includes neighboring sections at the respective β-strands or even whole β-strands (e.g., strands D and G). At pH ≈ 4 (simulation B4), the RMSF values are slightly increased in comparison to the other simulations. However, the characteristic regions, depicted in Figure 2, were retained.

Similarly, the CH3 domains have characteristic regions between residues 347–364, 372–381, 382–407, 408–424 and 427–437 (Figure 2). These characteristic regions remain clearly identifiable in all performed simulations and overlap with the *C*-terminal loop regions of the CH3 domains, *i.e.*, residues 358–362, (AB-loop, *i.e.*, connecting strands A and B), residues 383–391 (CD-loop) and residue 413–422 (EF-loop), which correspond to the positions where antigen binding sites are intended to be implemented in the design of Fcabs. At pH ≈ 4 (B4), the RMSF values increased but the distinctive fingerprint for the CH3 domain remained unchanged.

The radius of gyration (rgyr) consistently shows an average value of 1.4–1.5 nm for the individual domains of the Fc fragment and an average value of 0.7–0.8 nm for the glycan structures. This observation applies to most simulations performed. However, the radius of gyration for a complete monomer, consisting of one CH2 and one CH3 domain, tends to decrease by 0.1–0.3 nm during all simulations, suggesting increased motion of the domains.

**Figure 3.**
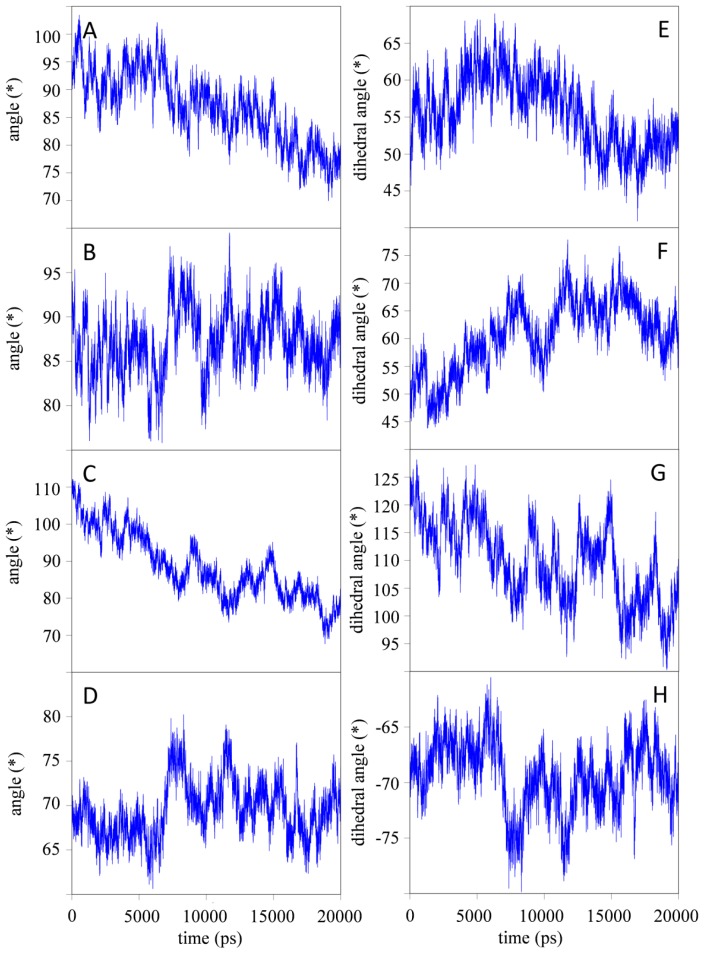
Relative orientation of individual domains as defined through the (dihedral) angles (in degree) as a function of time for simulation H7. (**A**) angle between *↛*_CH2A_, *↛*_CH3A_; (**B**) angle between *↛*_CH2B_, *↛*_CH3B_; (**C**) angle between *↛*_CH2A_, *↛*_CH2B_; (**D**) angle between *↛*_CH3A_, *↛*_CH3B_; (**E**) dihedral angle defined by *↛*_CH2A_, *⇉*_CH3A_, – *⇉*_CH2A_, *↛*_CH3A_; (**F**) dihedral angle defined by *↛*_CH2B_, *⇉*_CH3B_, – *⇉*_CH2B_, *↛*_CH3B_; (**G**) dihedral angle defined by *↛*_CH2A_, *⇉*_CH2B_, – *⇉*_CH2A_, *↛*_CH2B_; and (**H**) dihedral angle defined by *↛*_CH3A_, *⇉*_CH3B_, – *⇉*_CH3A_, *↛*_CH3B_. See Experimental Section and Supplementary Information for details concerning the definition of the vectors.

For all simulations, the relative orientations of the individual domains are depicted in Figure 3 (simulation H7) and Figures S6–S9 (simulation B4, B7, H4 and Q347E; see Supplementary Information). These measures appear quite noisy, but in general seem to fluctuate around stable values after the first 10 ns of simulation. For all simulations, the orientation of the CH2 domains with respect to each other seems to change significantly (Figure 3C,G). At low pH, the CH2 domains seem to reorient themselves with respect to the CH3 domains, while this effect is less pronounced for the simulations at neutral pH. Due to the large domain motion observed in simulation B4, the domains are able to interact with periodic copies of the other domains from time to time, with a maximum duration of 500 ps (*i.e.*, 2.5% of the simulation time). In all other simulations, such interactions occur rarely and for even shorter time intervals. Considering the short times during which such unphysical interactions over the periodicity occur, it will rather be the effect than the cause of the domain motions that are observed in the simulations, and have a negligible influence on the data reported here.

A molecular dynamics simulation is never finished, and one can never be 100% certain that additional conformations will not be observed if a simulation is extended. The current 20 ns simulations indicate that the individual domains remain stable, while some domain motion remains on a 2–4 ns timescale. The properties that are analyzed in more detail below, dihedral angle transitions, occurrence of hydrogen bonds and the contributions to the solvent accessible surface area, typically converge and relax on the sub-nanosecond timescale and proper ensemble averages can be calculated from these simulations.

#### 2.1.2. Impact of the Hinge Region and Dynamics of the Glycan Structure

Upon implementation of the hinge region *N*-terminally to the CH2 domains, the observed fluctuations in IgG1-Fc at pH ≈ 4 (simulation H4) and pH ≈ 7 (simulation H7) slightly decreased, suggesting that the hinge region has a slight stabilizing effect on the domain motion observed in the simulations (see previous section). Interestingly, the motion of the CH2 domains with respect to each other was only slightly influenced by inclusion of the hinge regions. This was seen both at pH ≈ 4 and pH ≈ 7 as well as in the variant (simulations H4, H7 and Q347E; Figure 3C and G, S69).

The glycan structures present in the simulations branch three times. The general trend in the simulation data is that the atoms towards the end of each branch have a slightly higher RMSF when compared to other atoms elsewhere in the glycan structure. Analysis of the number of transitions of dihedral angles between the energy minima as defined by the torsional potential energy term in the force field shows different dynamic behavior for the glycan structures in the different simulations. Table 1 shows the transitions for those dihedral angles along the bonds connecting monosaccharides that differ between simulations. While the number of dihedral angle transitions seems to increase upon inclusion of the hinge region at pH ≈ 7, the stabilizing mutation in the CH3-domain (Q347E) leads to a strikingly lower number of dihedral angle transitions in the glycan structures.

#### 2.1.3. Stabilizing Interactions

**Figure 4.**
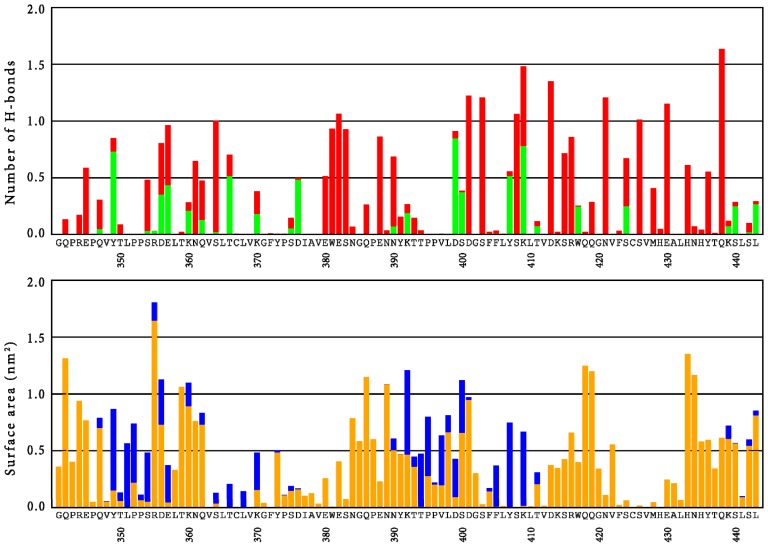
*Top panel*: Average number of hydrogen bonds (excluding backbone-backbone interactions) per residue of the CH3 domain, as observed in simulation H7. *The green fraction of the bars* indicate hydrogen bonds between chains A and B. *Bottom panel*: Average surface area per residue of the CH3 domain, as observed in simulation H7. *Orange bars* indicate solvent accessible surface area; *blue bars* indicate interfacial area between chains A and B.

A detailed analysis of the role of individual amino acid side chains in the CH3 domain of simulation H7 is presented in Figure 4. Here, the average amount of hydrogen bonds that involve side-chains is given, together with the surface area of the amino acids. The different colors in the bars categorize interface interactions in solvent accessible regions in the protein and intramolecular hydrogen bonds. This figure can be used to rationalize experimental data and point at potential sites where mutations may be most likely to improve the properties of the protein. For instance, a clear correlation is observed between the solvent accessible surface area of the residues and the amount of mutations that are observed in a random mutagenesis approach, screening for stability [35]. That is, the amino acids on the surface are more susceptible to stabilizing point mutations than amino acids in the core of the protein.

Experimentally, various mutations were explicitly shown to increase the stability of the CH3 domain by differential scanning calorimetry (DSC) [30]. Most notable are mutations Q347E, K360E/Q, Q418L and Q438K. Figure 4 shows that the latter position (Q438) contributes only moderately to the solvent-accessible surface area, while it is strongly involved in intramolecular hydrogen bonds. Replacing the polar amino acid with a fully charged one potentially increases these interactions even further. For the first three positions (Q347, K360 and Q418), only moderate intramolecular hydrogen bonds were observed in the wild-type, while their contributions to the solvent accessible surface area (SASA) are considerable. The mutations are expected to lead to changes in the network of hydrogen bonds and salt bridges at the surface of the protein (Q347E, K360E/Q) or to enhance hydrophobic interactions towards the core of the protein (Q418L). The mutation Q347E at the CH3 domains was studied in more detail by explicit simulations of the mutant. The mutation led to a local stabilization of the side-chain, as reflected by significantly reduced RMSF-values of the side-chain atoms. However, an overall stabilizing effect on entire domains is only observed for the CH3 domain in chain B, while an increase in average RMSF-values is observed for the CH3 domain in chain A (see also Table S1). The analysis of hydrogen bonds is summarized in Table 2. In comparison to simulation H7, IgG1-Fc in the Q347E simulation shows more protein-to-glycan hydrogen bonds and a similar number of protein-to-solvent or glycan-to-solvent hydrogen bonds. Simulation Q347E also shows less glycan-to-glycan hydrogen bonds. The stabilizing single point mutation leads to four times more intra-chain hydrogen bonds between this residue and the neighboring tyrosine residue of the same chain, Y349, when compared to the wild-type residue. This reduces the number of inter-chain hydrogen bonds that Y349 can form with D356 and E357 of the other chain. Protonation similarly decreases the interaction between D356 and E357 with Y349 (simulations B4 and H4). In comparison to wild-type Fc protein, in the variant Q347E the residues D356 and E357 do not form alternative inter-chain bonds either, suggesting that the interaction between chains A and B is reduced. No additional ionic interactions in the mutant were observed.

Another interesting feature of the Fc fragment is the tyrosine-tyrosine stacking that occurs at the interface between two CH3 domains involving Y407 of both chains (Figure 1). The average distances and angles between the aromatic rings (Table S2) suggest that this tyrosine-tyrosine stacking remains intact for the entire length of any simulation. The distance between the two Y407 side chains is shortest for simulation H7 and is slightly increased in simulation Q347E.

### 2.2. Discussion

Engineering the human IgG1-Fc for introduction of antigen binding sites as well as recombinant production of potential future therapeutic Fcabs needs more information about the conformational stability and local flexibility of the immunoglobulin domains as well as the cross-talk between the CH2 and CH3 domains of this homodimeric protein. In fact, one recent focus in the development of novel therapeutic antibodies is the creation of monomeric Fc fragments [25] or even single monomeric CH3 domains [26]. For the latter approach, mutations at four positions were proposed to destabilize the dimerization: L351, T366, L468 and P395 [26]. Figure 4 readily explains why these positions seem appropriate; these amino acids all contribute to the dimer interface area, while position T366 is also involved in an interfacial hydrogen bond interaction. Various other positions might be identified for which a mutation could potentially reduce the hydrophobic surface (e.g., F405) or could disrupt hydrogen bond interactions at the interface (e.g., Y349, D356, E357, D376, D399, S400 or Y407). On the other hand, residues S354 or K409, which also contribute to the interface surface, would be inappropriate choices, as these are involved in significant intra-chain hydrogen bonds. Combining these suggestions with the experimentally determined stability landscape [35] suggests that, in particular, D376 and S400 are residues for which mutations can be expected to lead to stable monomeric CH3 domains.

Wang *et al*. [33] performed molecular dynamics simulations of full-size glycosylated and deglycosylated murine IgG2a mAb and some specific fragments of it. The simulations of glycosylated crystallizable fragments of IgG1 in this study have comparable RMSD values and follow similar tendencies. The RMSF also shows a similar trend, but the values reported here are two- to three-fold lower, which may be explained by differences in the simulation setup, the force field and the fact that only the Fc protein is simulated. Additionally, the sequence identity of the respective crystallizable fragments of IgG1 and IgG2a is only 64%. Although a direct comparison between results from the IgG1-Fc fragment and the IgG2a-Fc is not straightforward, the characteristic regions found in the RMSF of CH2 and CH3 domains seem to be conserved, especially those of high fluctuations including the *C*-terminal AB-, CD- and EF-loops at the CH3 domains, *i.e.*, the engineering sites for the design of Fcabs. The SASA of the three loops in the dimeric structure amounts to 17.7 ± 1.1 nm^2^ per monomer for the simulations without the hinge region (B4 and B7), while it is 14.5 ± 1.0 nm^2^ for the systems in which the hinge region was included (H4, H7 and Q347E). This surface area is potentially available for antigen binding through the CH3 interface, while only 0.4 nm^2^ of the loop surface is involved in the interface between the monomers. The loops of one monomer do not seem to interact significantly with the loops of the other monomer and, therefore, the individual contribution to the antigen binding site seems to be independent.

Figure 4 allows us to suggest positions in the loop regions for which mutations that modify the binding characteristics are most likely allowed in terms of protein structure [29]. In the AB-loop, amino acids T359 and K360 seem to contribute significantly to the solvent accessible surface area, while they are not involved in hydrogen bonding interactions that are too strong and which may be relevant for stability of the CH3 domain itself. In agreement with this, the experimentally determined stability landscape suggests that these residues are susceptible to stabilizing mutations [35]. Furthermore, stability measurements on libraries, in which these positions are randomized, confirm these findings [36]. In the CD-loop, our simulations suggest that positions N384, P387 and N389 are potential mutation candidates which are confirmed by the stability measurements on randomized libraries. The strong hydrogen bond of E388 offers a rationalization for the destabilizing effect that was observed when this position was randomized [36]. Finally, for the EF-loop, the joint insight from experiments and our simulations suggest that residues K414, Q418 and Q419 are the most promising candidates to change the functionality of the CH3 domain, without losing its stability, while the interactions of R416 and W417 rather confirm that these residues are relevant for stability. As discussed above, it was independently shown that mutation Q418L increases the melting temperature of this domain by 3.7 °C [30].

Kortkhonjia *et al*. [34] found in their simulations using the mutual information metric from information theory that domain movement in IgGs is only horizontally correlated *i.e.*, CH1 domain with CH1 domain, CH2 with CH2, and CH3 with CH3. In the current simulations, we observe that the RMSD and RMSF of individual domains are relatively stable, but domain motions do occur, independently of the presence of the hinge region (see Figures 3 and S6–S9 in Supplementary Information). Altogether, the trend seems to show that simulating fragments (e.g., IgG1-Fc) may be sufficient when one is interested in effects and changes localized in that fragment. Simulating a single domain (e.g., CH3) for similar purposes could also be considered, although effects on the interactions between domains were observed.

As explained in the introduction, complete homodimeric Fc proteins are of interest as scaffolds for the design of monoclonal therapeutic antibodies. The results from the radius of gyration (rgyr) show that the individual CH2 and CH3 domains and glycans remain stable during the simulations, while the rgyr of complete monomers converge to a smaller value during the simulation time of 20 ns. The CH2 and CH3 domains mainly consist of β-sheets that are stabilized by a large number of hydrogen bonds, and large structural changes in the secondary structure are not observed. However, monomeric Fc consisting of two domains shows a larger reduction in rgyr which could be attributed to the CH2 and CH3 domains approaching each other during the simulations. This is supported by the data presented in Figure 3A,E which shows a decrease of the angle between the normal modes of the CH2 and CH3 domains.

The hinge region that connects *N*-terminally the CH2 domains of two monomers has been shown to move independently from other parts of a full mAb [33,34]. The current data on IgG1-Fc comparing simulations B7 and H7 suggest that the presence of the hinge region does not significantly affect the overall domain motion or stability of the interactions between the domains. A larger effect on the domain motions was observed when the pH was lowered from pH ≈ 7 to pH ≈ 4, resulting in more significant changes in the orientation of the CH2 domain with respect to the CH3 domain.

The glycan structure had a rather stable rgyr, while the terminal glycan structures show significant conformational freedom (Table 1). This seems to suggest that the glycan structures are relatively freely moving between the CH2 domains, a phenomenon that has also been observed experimentally [37]. Kortkhonjia *et al*. [34] found that the glycans were essential to the interface between the CH2 domains. Interestingly, our simulations indicate that the glycans form more hydrogen bonds with the individual protein chains at the cost of glycan-glycan interactions. This was more pronounced in the simulation Q347E. The increased glycan to protein interactions, in turn, reduce the number of dihedral angle transitions observed for the terminal glycan groups in this simulation (see Table 1).

The single mutant Q347E was selected for this MD investigation since DSC investigations showed that the midterm transition (*T*_m_ value) for the CH3 domain was shifted from 78 (wild-type IgG1-Fc) to 81 °C (Q347E) [30]. Based on the RMSF values in Table S1, the CH3 domain as a whole seems to be stabilized in chain B, but destabilized in chain A as observed by an increase in fluctuations. The hydrogen-bond analysis in Table 2 offers a molecular interpretation of the experimental observations. Upon mutation, residue 347 forms significantly more intra-domain hydrogen bonds with Y349, leading to a reduced flexibility of the side chain itself and possibly explaining the increased stability of the CH3 domain. In simulations of wild-type IgG1-Fc, Y349 was involved in inter-domain hydrogen bonds with D356 and E357 of the other CH3 domain. These hydrogen bonds are significantly weakened in the mutant and no additional inter-domain hydrogen bonds are formed. Thus, the simulations suggest that the exchange of Q347 to E347 has a stabilizing effect on the CH3 domain, but a destabilizing effect on the interactions between the domains. This observation is extended to the interactions between the glycan structures, which were previously postulated to be important for the inter-chain stability [34] and which seems to be reduced in simulation Q347E, although a direct causality cannot be determined.

## 3. Experimental Section

### 3.1. Simulation Setup

The GROMOS11 package (BIOMOS B.V., Groningen, The Netherlands) for biomolecular simulations [38] was used for all MD simulations. The GROMOS++ software (BIOMOS B.V.) for analysis of biomolecular trajectories was used to assist in setting up the simulations [39]. Five systems were created based on the crystal structure with PDB (RCSB Protein Data Bank. RCSB-Rutgers, Piscataway, NJ, USA; RCSB-SDSC, La Jolla, CA, USA; RCSB-BMRB, Madison, WI, USA) ID 1OQO. Two systems represent the protonation state of 1OQO corresponding to pH ≈ 4 (referred to as B4) and pH ≈ 7 (referred to as B7). Two additional systems of 1OQO were prepared with the hinge region from the full-size human IgG1 crystal structure with PDB ID 1HZH [40] modeled in, again at protonation states corresponding to pH ≈ 4 (referred to as H4) and pH ≈ 7 (referred to as H7). In simulations B4 and H4, all aspartic and glutamic acids were protonated, while the deprotonated amino acids were used to describe simulations B7 and H7. Finally, one system was prepared based on system H7 in which a mutation of Q347 to E347 (referred to as Q347E) was introduced in both chain A and chain B.

Force-field parameters for the protein were taken from the GROMOS 54A7 united-atom force field [41]. Force-field parameters for the glycans were taken from the GROMOS 56A_CARBO_ united-atom force field [42]. The hinge region was modeled into 1OQO in two steps. First, the IgG1-Fc and the hinge regions of 1HZH were structurally aligned to 1OQO. For each chain, the coordinates corresponding to the 12 residues preceding the first commonly shared atom between 1HZH and the *N*-terminus of 1OQO were transferred from 1HZH to 1OQO. The resulting structure was optimized and relaxed by decreasing the force constant of an imposed position restraint on all solute atoms of 1OQO from 2.5 × 10^4^ to 0 kJ·mol^−1^·nm^2^ in 5 discrete simulation steps of 20 ps at 300 K. No position restraints were imposed on any atoms that were transferred from 1HZH.

The glycan tree structures were constructed by linking monosaccharides in the order as specified in Figure 1. Detailed topological information of the glycan tree structures is provided in Figure S10 in the Supplementary Information. Complete building blocks for the glycans are also available in the Supplementary Information (Figures S12–S19).

A rectangular simulation box with a minimal solute to wall distance of 1.0 nm was used and simple point charge (SPC) water molecules [43] were added. To neutralize the net-charges of the systems at pH ≈ 4 (amounting to ~50 *e*), Na^+^ and Cl^−^ ions were added to the simulation boxes of simulations B4 and H4 (with an excess of Cl^−^). The systems at pH ≈ 7 are roughly neutral, and no counter ions were necessary. Dimensions and the detailed composition of all simulations are available in Table S3 of the Supplementary Information.

Velocities corresponding to an initial temperature of 60 K were randomly assigned from a Maxwell-Boltzmann distribution to all atoms before the thermalization process of each simulation, during which systems were heated to the desired temperature through gradual increase of temperature (Δ*T* = 60 K) while simultaneously decreasing the force constant of an imposed position restraint on all solute atoms from 2.5 × 10^4^ to 0 kJ·mol^−1^·nm^−2^ in 5 discrete simulation steps of 20 ps each.

Simulations were performed with a constant number of particles, at a constant pressure of 1 atm and at a constant temperature of 300 K. Each simulation ran for an additional 20 ns after thermalization. Solvent and solute degrees of freedom were coupled separately to two temperature baths with a relaxation time of 0.1 ps using the weak-coupling method [44]. Pressure was also kept constant using the weak-coupling scheme, with a relaxation time of 0.5 ps and an estimated isothermal compressibility of 4.575 × 10^−4^ (kJ·mol^−1^·nm^−3^)^−1^. The leap-frog algorithm [45] with a timestep of 2 fs was used. All bonds were constrained to their minimum energy values using the SHAKE algorithm [46]. Center of mass translation was removed every 1000 steps. Non-bonded interactions were calculated using a triple range cut-off scheme. Interactions up to a short-range distance of 0.8 nm were calculated at every timestep from a pairlist that was updated every 5 steps. At pairlist construction [47], interactions up to an intermediate range of 1.4 nm were also calculated and kept constant between updates. A reaction field contribution [48] was added to the forces and energies to account for a dielectric continuum with relative permittivity of 61 beyond the cut-off sphere of 1.4 nm [49]. Non-bonded solvent-solvent interactions were calculated using the graphics processing unit (GPU) solvent loop implemented in GROMOS11, which speeds up calculations significantly [50].

### 3.2. Data Analysis

Only coordinate trajectories generated after the thermalization were used for analysis. Starting from the first frame of a trajectory, coordinates that are separated by a time interval of 2 ps were included. The GROMOS++ software for analysis of biomolecular simulation trajectories [39] was used for analysis.

The atom-positional root-mean-square deviations (RMSD) of backbone atoms C, CA and N of individual domains were calculated as a function of time. RMSD values were calculated with respect to the first set of coordinates in a trajectory after a roto-translational fit involving a selection of atoms, individual domains or combinations thereof. The atom-positional root-mean-square fluctuation (RMSF) of selected atoms was calculated after a similar roto-translational fit.

Hydrogen-bond analyses were performed using as geometric criteria a maximum hydrogen-acceptor distance of 0.25 nm and a minimum donor-hydrogen-acceptor angle of 135 degree. Each hydrogen bond between a pair of distinct atoms was considered unique and the prevalence of a hydrogen bond was determined as the percentage of time during which the hydrogen bond was observed in the simulation. Prevalence of hydrogen bonds between residue and domains is calculated as the sum of the occurrence of hydrogen bonds involving any atom in the residue or domain.

Dihedral-angle torsional profiles exhibit more than one optimal value and transitions may occur during MD simulations. The number of dihedral angle transitions between energy minima was calculated for all dihedral angles linking the individual moieties in the glycan structures. A transition was counted whenever the value of the dihedral angle crossed a maximum in the potential energy as defined by the torsional angle term in the force field between subsequent snapshots in the trajectory.

The orientation of two domains with respect to each other can be described using angles and dihedral angles. Representative angles and dihedral angles were calculated from the positions of the domains as given by the center of geometry (*⇉_domain_*) and from the largest principle component of the domain (*↛_domain_*) calculated as the eigenvector with the largest eigenvalue of the covariance matrix of atomic positions [51]. Definition of angles and dihedral angles are given in Supplementary Information, Figure S11 and Table S4. Possible interactions between periodic copies of the solute were determined by subtracting the distance between a solute atom in the simulation box and any solute atoms in any periodic copy of the simulation box from the long-range cut-off value of 1.4 nm. Interactions over the periodicity were observed if the resulting value is smaller than 0 nm.

The radius of gyration (rgyr) was calculated for the complete systems as well as for the individual domains and the glycans.

Solvent accessible surface areas (SASA) of the systems were calculated by rolling a water-sized probe (with a radius of 0.14 nm) over the surface of the protein and keeping track of individual atomic contributions. By performing this analysis separately for the monomers and the dimeric structures an estimate of the surface interface was obtained.

## 4. Conclusions

Molecular dynamics simulations on the human crystallizable fragment of immunoglobulin G1 were performed with and without the hinge region, and at pH ≈ 4 and pH ≈ 7. Overall, the structures of the individual domains were well maintained, while some domain motion, which might be pH-dependent, was observed, in particular at lower pH. Insight into the potential effects of various mutations on fragment stability or binding characteristics was obtained from analyses of interactions and accessibilities as observed in the various molecular dynamics simulations performed. In particular, the effect of mutation Q347E was studied, offering a rationale for the increase in stability which was observed in DSC measurements.

Overall, our work exemplifies how molecular dynamics simulations can support the characterization and design of novel pharmaceutically relevant proteins.

## Supplementary Information



## Figures and Tables

**Table 1.** Dihedral angle transitions in the glycan tree which show a significantly higher number of transitions between energy minima.

Dihedrals	Atoms	Residue *	Chain	B7	H7	Q347E	B4	H4
3	O5-C5-C6-O6	1–6	A	86	99	23	1	6
			B	60	115	11	91	23

9	C4-O4-C1-O5	2–3	A	12	0	128	2346	748
			B	2	1435	50	0	12

10	C2-O2-C1-O5	4–5	A	70	320	18	44	0
			B	892	418	140	236	28

18	C2-O2-C1-O5	7–8	A	98	417	4	2	40
			B	4	617	2	54	229

* Corresponding residue numbers of glycans according to the numbering in Figure 1.

**Table 2.** Prevalence of hydrogen bonds of selected residues (wild-type, mutant residue and neighboring residues, glycans, solvent) at different protonation states and with/without the hinge region.

From a	To a	B7 (%)	H7 (%)	Q347E (%)	B4 (%)	H4 (%)
Q/E347A	Y349A	21	11	89	23	21
Y349A	D356B	20	43	22	14	12
	E357B	6	18	1	0	0
Q/E347A	solvent	0	9	7	1	0
D356A	chain B	77	20	16	16	6
E357A	chain B	4	63	1	0	3
Q/E347B	Y349B	16	14	95	18	5
Y349B	D356A	53	10	7	10	3
	E357A	1	36	0	0	2
Q/E347B	solvent	29	19	14	5	4
D356B	chain A	31	49	24	45	20
E357B	chain A	15	22	4	0	1

chain A	chain B	611	640	391	456	405
chain A	glycans	471	417	529	438	475
chain B	glycans	482	370	680	396	448
glycans	glycans	661	622	497	636	642
chain A	solvent	4220	9889	9630	1318	6998
chain B	solvent	10083	7714	8944	7498	5964
glycans	solvent	2178	2606	2771	1091	2235

a The letter before the residue number specifies the residue name and the letter after the residue number specifies the chain.
