# Supplementary material for: Molecular Dynamics Simulation of the Crystallizable Fragment of IgG1—Insights for the Design of Fcabs
Source: Int J Mol Sci. 2014 Jan 2;15(1):438–55. doi: 10.3390/ijms15010438 (PMC3907818; doi:10.3390/ijms15010438)
Supplement: Supplementary file 1 [file ijms-15-00438-s001.pdf]

Supplementary Information

Contents

1. Tables and Figures......S2
2. Building Blocks and Topologies......S13
2.1. *Atom Numbering Overview*......S13
2.2. *α-*L*-Fucose (FUC) Building Block*......S14
2.3. *β-*L*-Fucose (FUL) Building Block*......S18
2.4. *β-*D*-Mannose (BMA) Building Block*......S24
2.5. *α-*D*-Mannose(MAN) Building Block*......S30
2.6. N-*Acetyl-*D*-Glucosamine (NAG) Building Block*......S36
2.7. *2-(Acetylamino)-2-Deoxy-A-*D*-Glucopyranose (NDG) Building Block*......S43

1. Tables and Figures

Table S1. Average protein backbone RMSF of wild-type and mutant Fc homodimer in nm.

Chain	Structure	B7	H7	Q347E	B4	H4
A	Q/E347 *	0.105	0.130	0.095	0.124	0.119
	CH2 domain	0.082	0.072	0.078	0.096	0.078
	CH3 domain	0.083	0.078	0.094	0.080	0.109
	Glycans	0.076	0.065	0.063	0.102	0.055
B	Q/E347 *	0.124	0.148	0.063	0.140	0.126
	CH2 domain	0.080	0.079	0.083	0.096	0.113
	CH3 domain	0.094	0.092	0.082	0.107	0.086
	Glycans	0.089	0.078	0.070	0.097	0.068

* These RMSF were calculated using all atoms in the residue.

Table S2. Distance and angle describing TYR407-TYR407 stacking at the CH3–CH3 interface.

Property	B7	H7	Q347E	B4	H4
Distance [a]	0.453 ± 0.24	0.420 ± 0.023	0.450 ± 0.027	0.459 ± 0.024	0.444 ± 0.34
Angle [b]	2.91 ± 0.12	2.91 ± 0.13	2.88 ± 0.14	2.89 ± 0.13	2.80 ± 0.19

[a] The calculated distance is the distance between the center of geometry of the aromatic ring of the two stacked TYR407; [b] The angle is calculated from a vector derived from two opposite atoms in the aromatic ring of the two stacked TYR407.

Table S3. Initial simulation box properties of simulation B4, B7, H4, H7 and Q347E.

System	X-Dimension (nm)	Y-Dimension (nm)	Z-Dimension (nm)	Na^+	Cl^-	H_2O
B4	7.33	8.29	9.19	28	81	15944
B7	7.31	8.29	9.19	0	0	16007
H4	8.19	8.66	10.17	48	108	20929
H7	8.25	8.82	10.16	0	0	20978
Q347E	8.53	9.19	10.42	0	0	24306

Table S4. Definition of (dihedral) angles used to define the orientation of domains.

Type	Code	Defining Vectors
Angle	A	$\vec{n}_{CH2A}, \vec{n}_{CH3A}$
	B	$\vec{n}_{CH2B}, \vec{n}_{CH3B}$
	C	$\vec{n}_{CH2A}, \vec{n}_{CH2B}$
	D	$\vec{n}_{CH3A}, \vec{n}_{CH3B}$
Dihedral	E	$\vec{n}_{CH2A}, \vec{r}_{CH3A} - \vec{r}_{CH2A}, \vec{n}_{CH3A}$
	F	$\vec{n}_{CH2B}, \vec{r}_{CH3B} - \vec{r}_{CH2B}, \vec{n}_{CH3B}$
	G	$\vec{n}_{CH2A}, \vec{r}_{CH2B} - \vec{r}_{CH2A}, \vec{n}_{CH2B}$
	H	$\vec{n}_{CH3A}, \vec{r}_{CH3B} - \vec{r}_{CH3A}, \vec{n}_{CH3B}$

Figure S1. Atom-positional root-mean-square deviations with respect to the initial structure for individual domains in simulation B4. (**A**) Chain A CH2 domain. (**B**) Chain B CH2 domain. (**C**) Chain A CH3 domain. (**D**) Chain B CH3 domain. (**E**) Chain A glycans. (**F**) Chain B glycans.

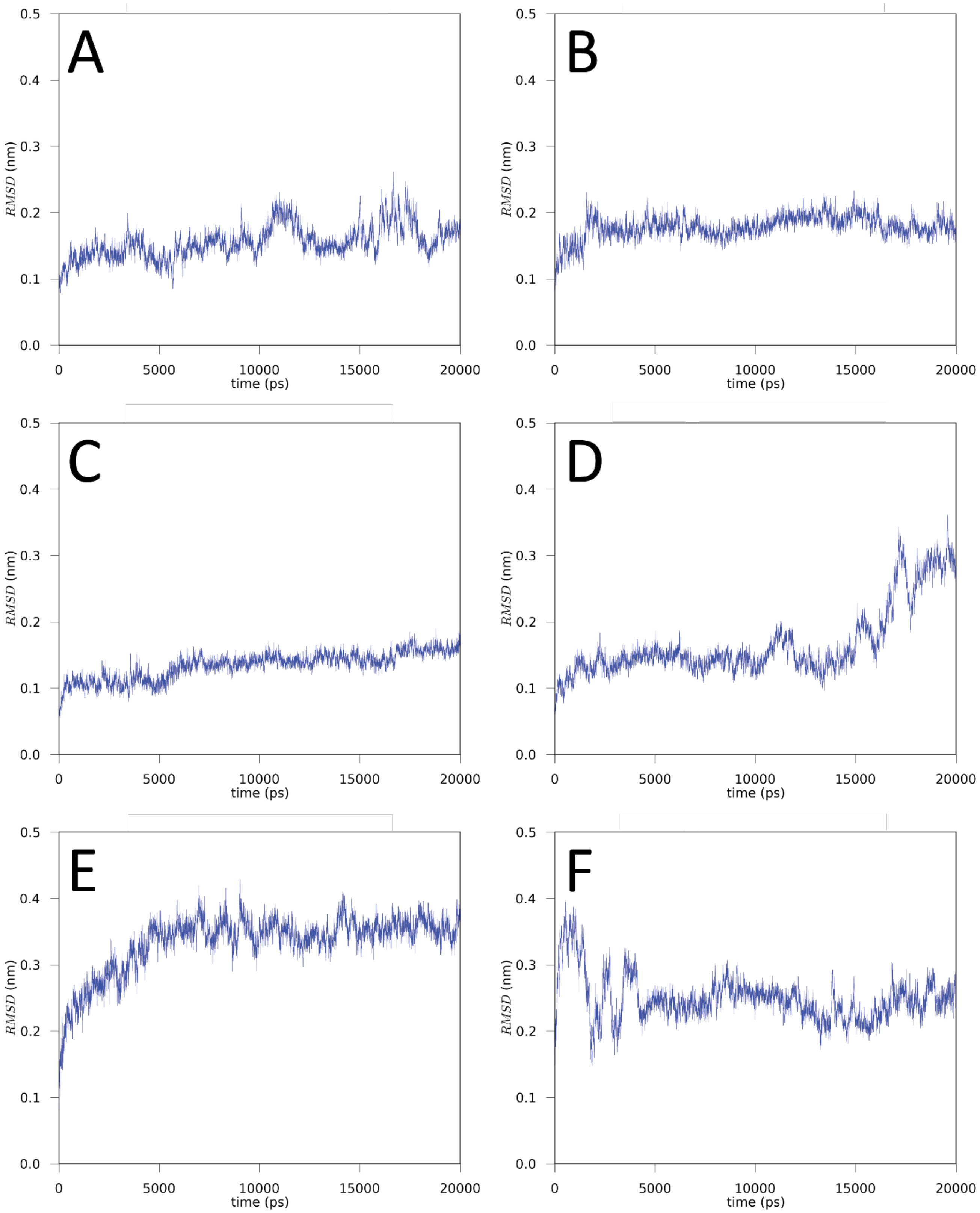

Figure S2. Atom-positional root-mean-square deviations with respect to the initial structure for individual domains in simulation B7. (**A**) Chain A CH2 domain. (**B**) Chain B CH2 domain. (**C**) Chain A CH3 domain. (**D**) Chain B CH3 domain. (**E**) Chain A glycans. (**F**) Chain B glycans.

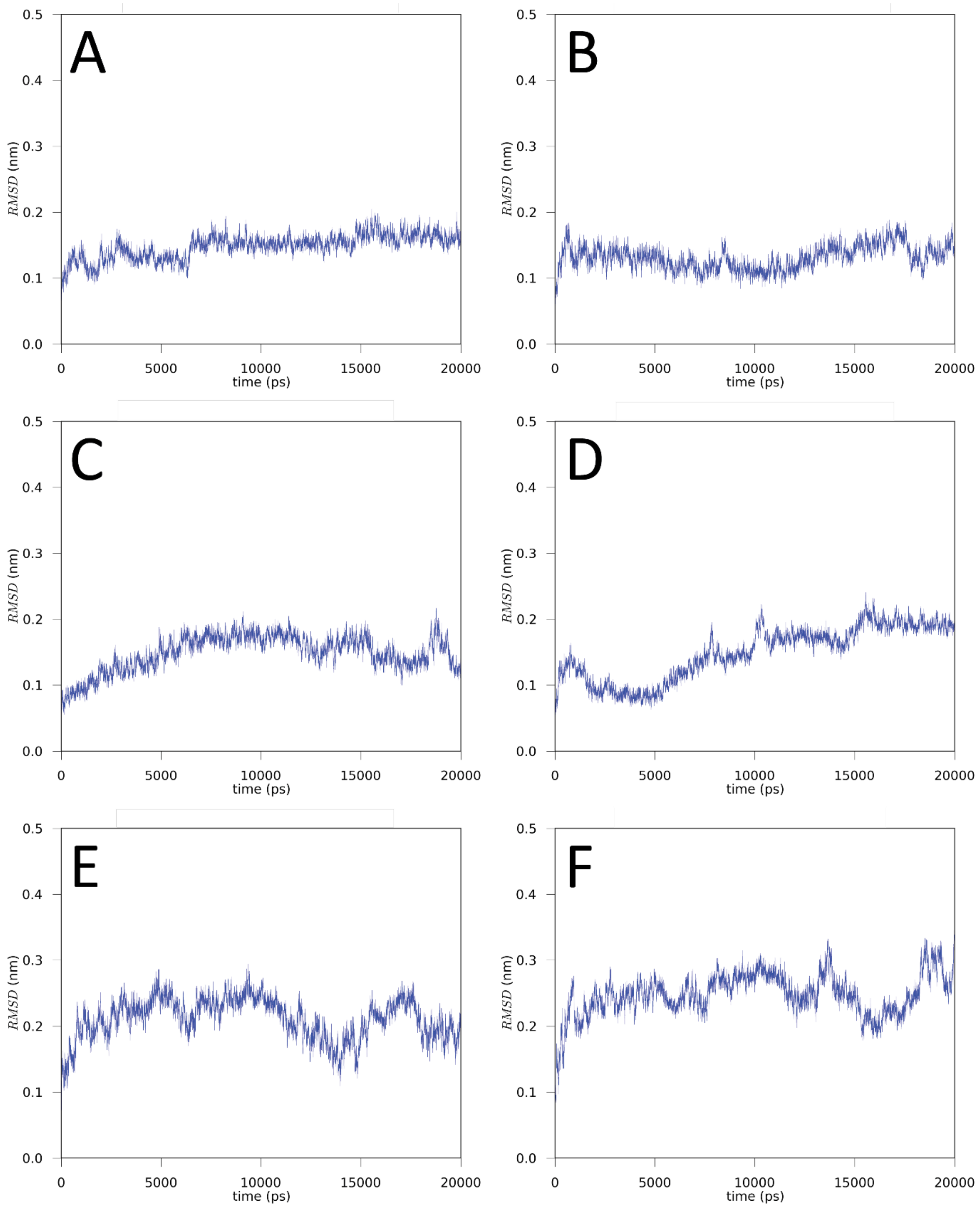

Figure S3. Atom-positional root-mean-square deviations with respect to the initial structure for individual domains in simulation H4. (**A**) Chain A CH2 domain. (**B**) Chain B CH2 domain. (**C**) Chain A CH3 domain. (**D**) Chain B CH3 domain. (**E**) Chain A glycans. (**F**) Chain B glycans.

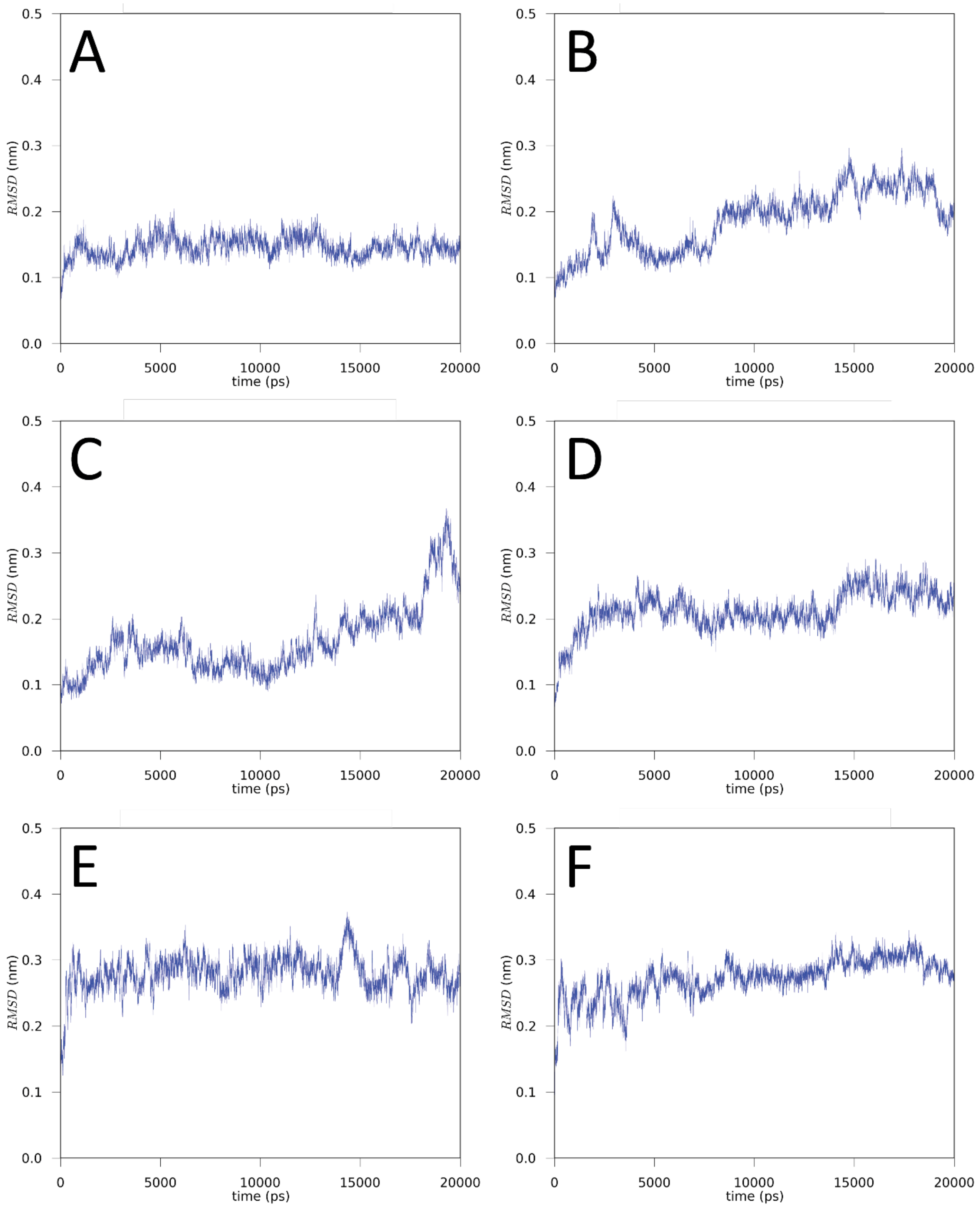

Figure S4. Atom-positional root-mean-square deviations with respect to the initial structure for individual domains in simulation H7. (**A**) Chain A CH2 domain. (**B**) Chain B CH2 domain. (**C**) Chain A CH3 domain. (**D**) Chain B CH3 domain. (**E**) Chain A glycans. (**F**) Chain B glycans.

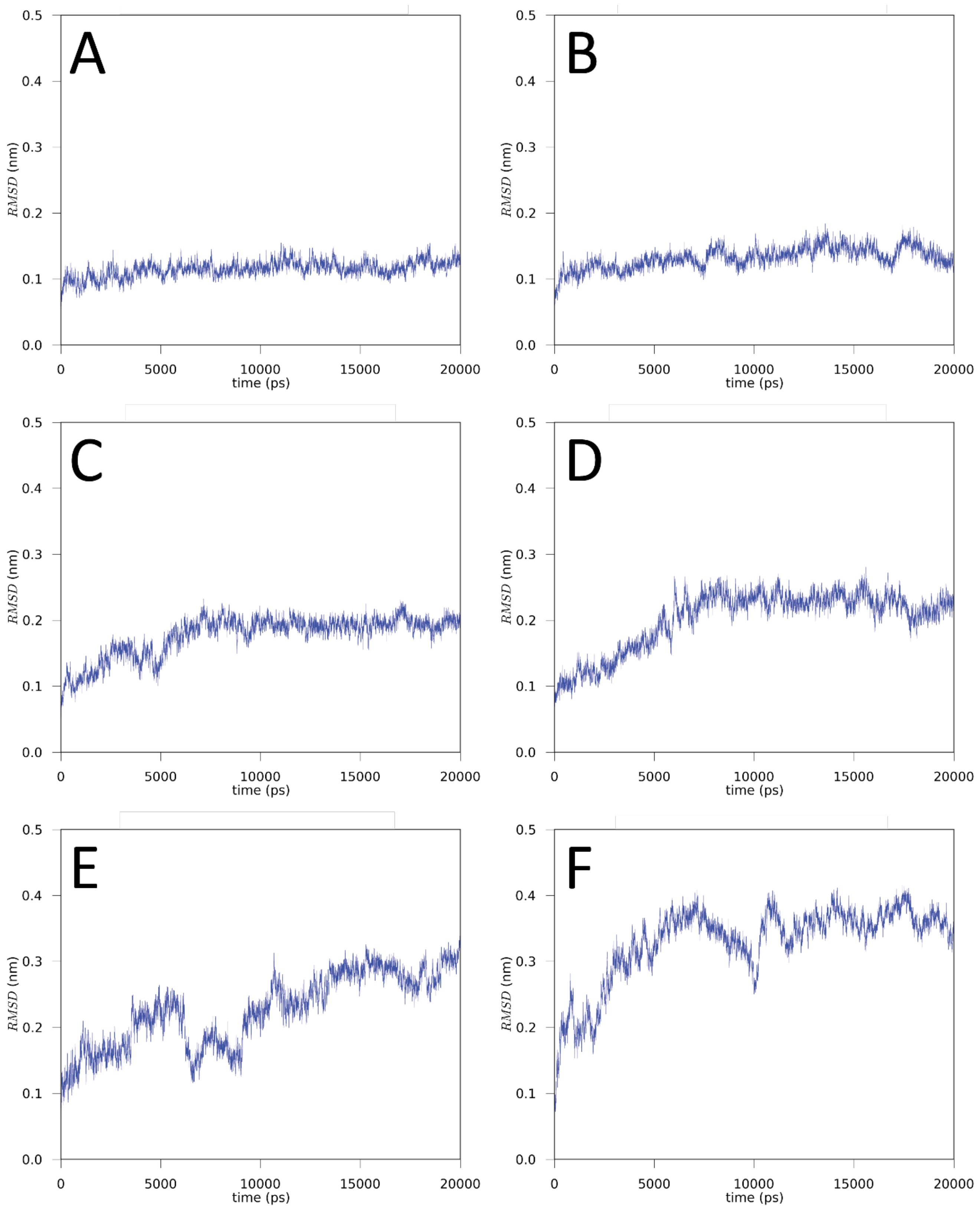

Figure S5. Atom-positional root-mean-square deviations with respect to the initial structure for individual domains in simulation Q347E. (**A**) Chain A CH2 domain. (**B**) Chain B CH2 domain. (**C**) Chain A CH3 domain. (**D**) Chain B CH3 domain. (**E**) Chain A glycans. (**F**) Chain B glycans.

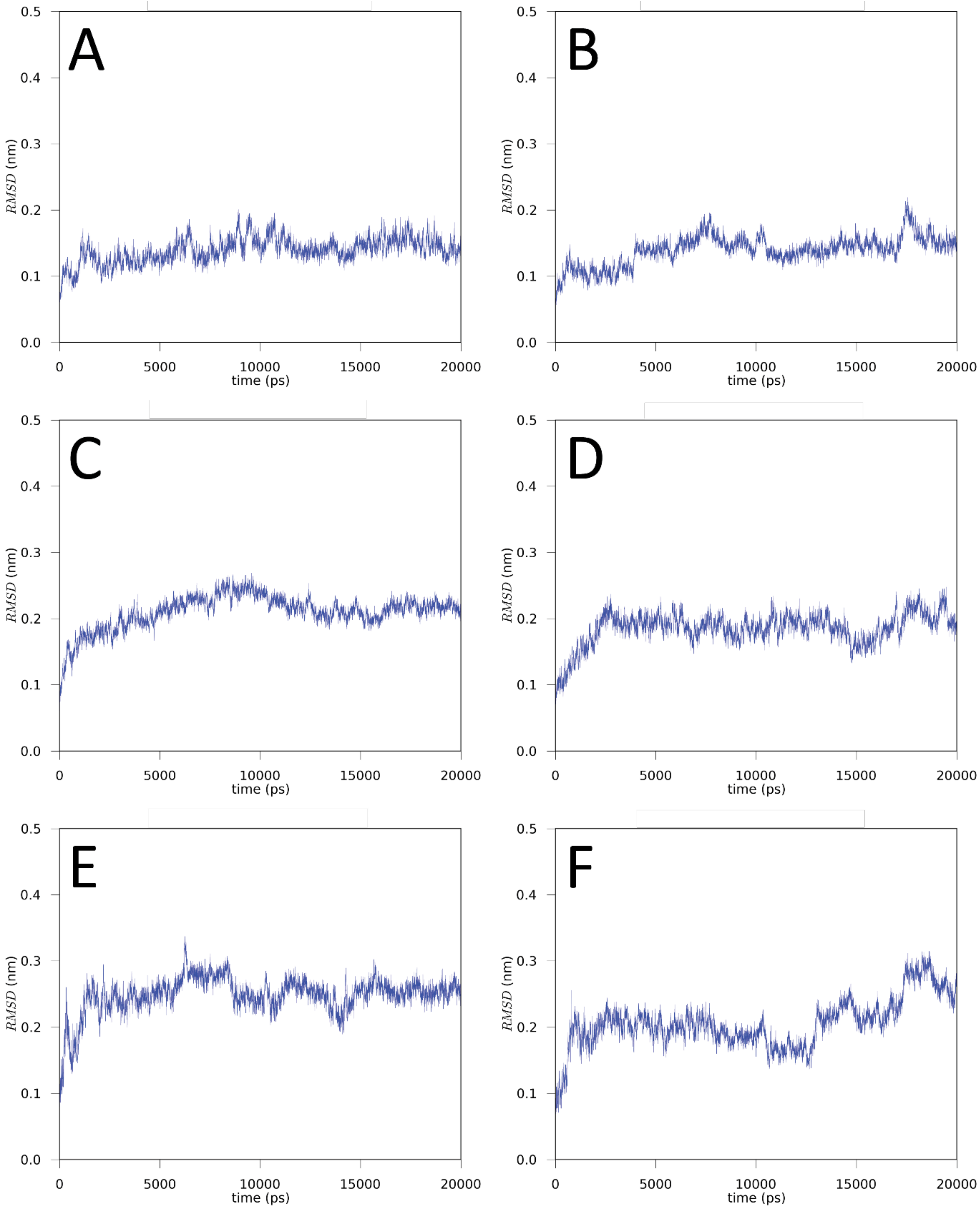

Figure S6. Relative orientation of individual domains as defined through the (dihedral) angles (in degree) in Table S4 as a function of time for simulation B4.

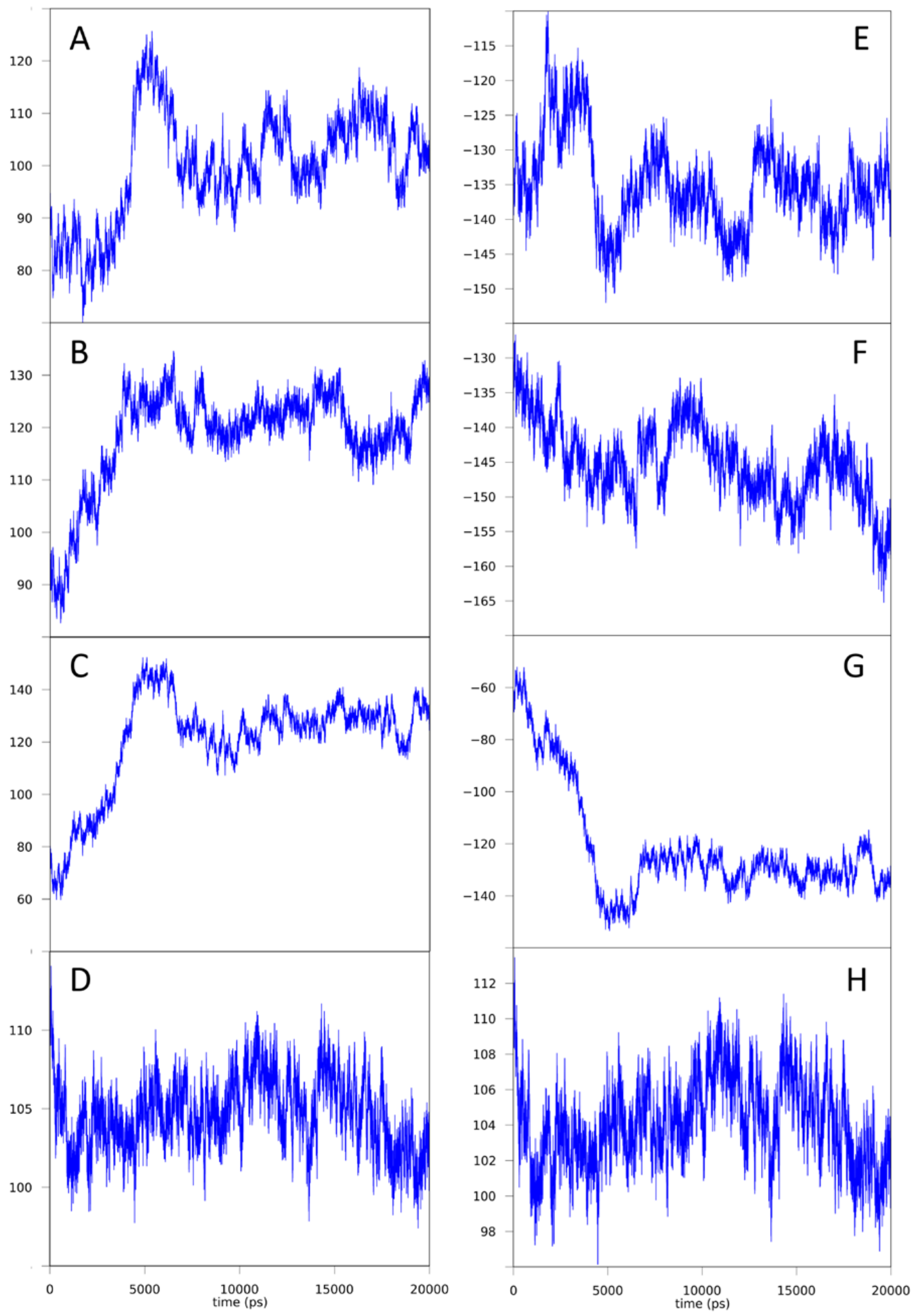

Figure S7. Relative orientation of individual domains as defined through the (dihedral) angles (in degree) in Table S4 as a function of time for simulation B7.

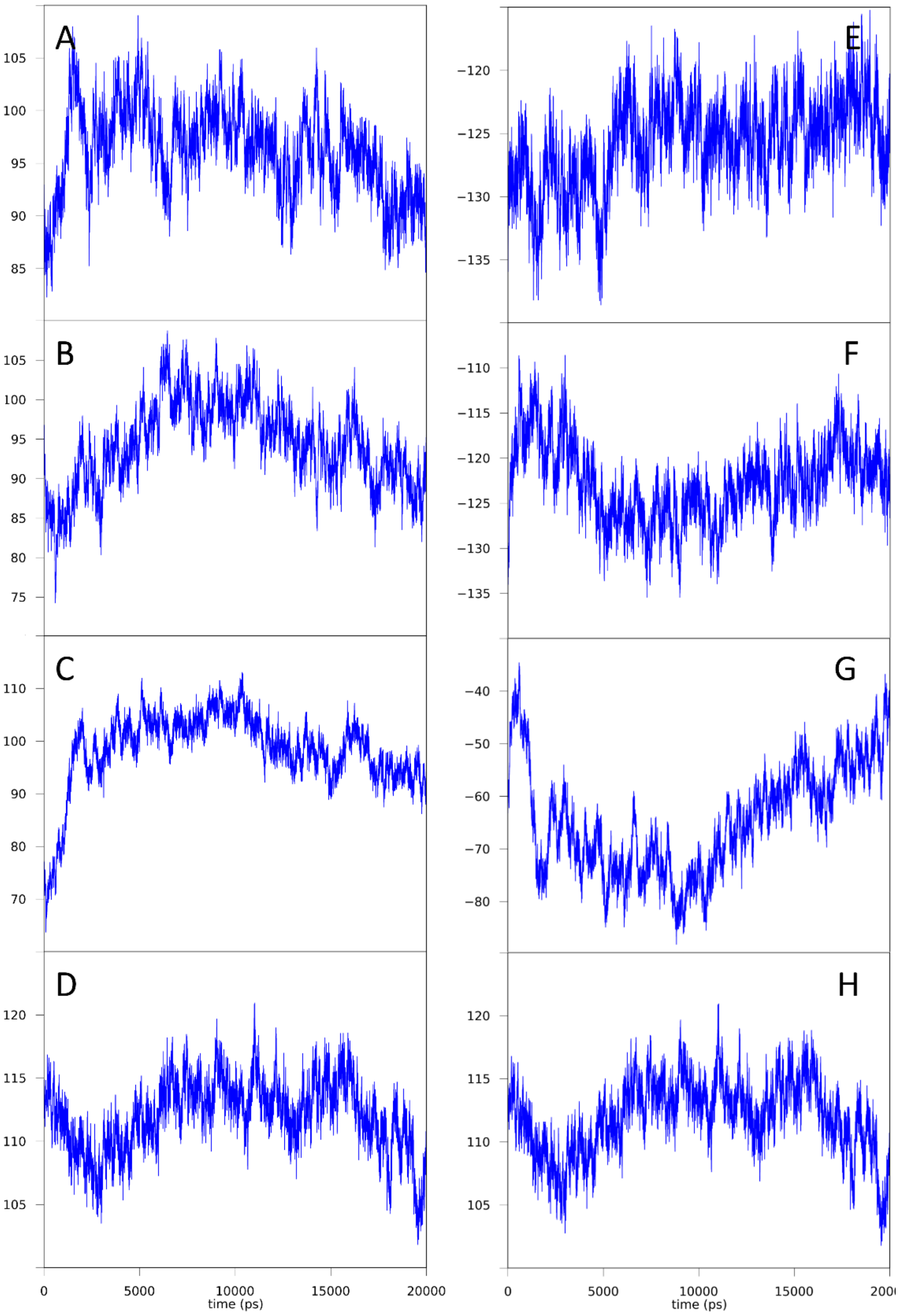

Figure S8. Relative orientation of individual domains as defined through the (dihedral) angles (in degree) in Table S4 as a function of time for simulation H4.

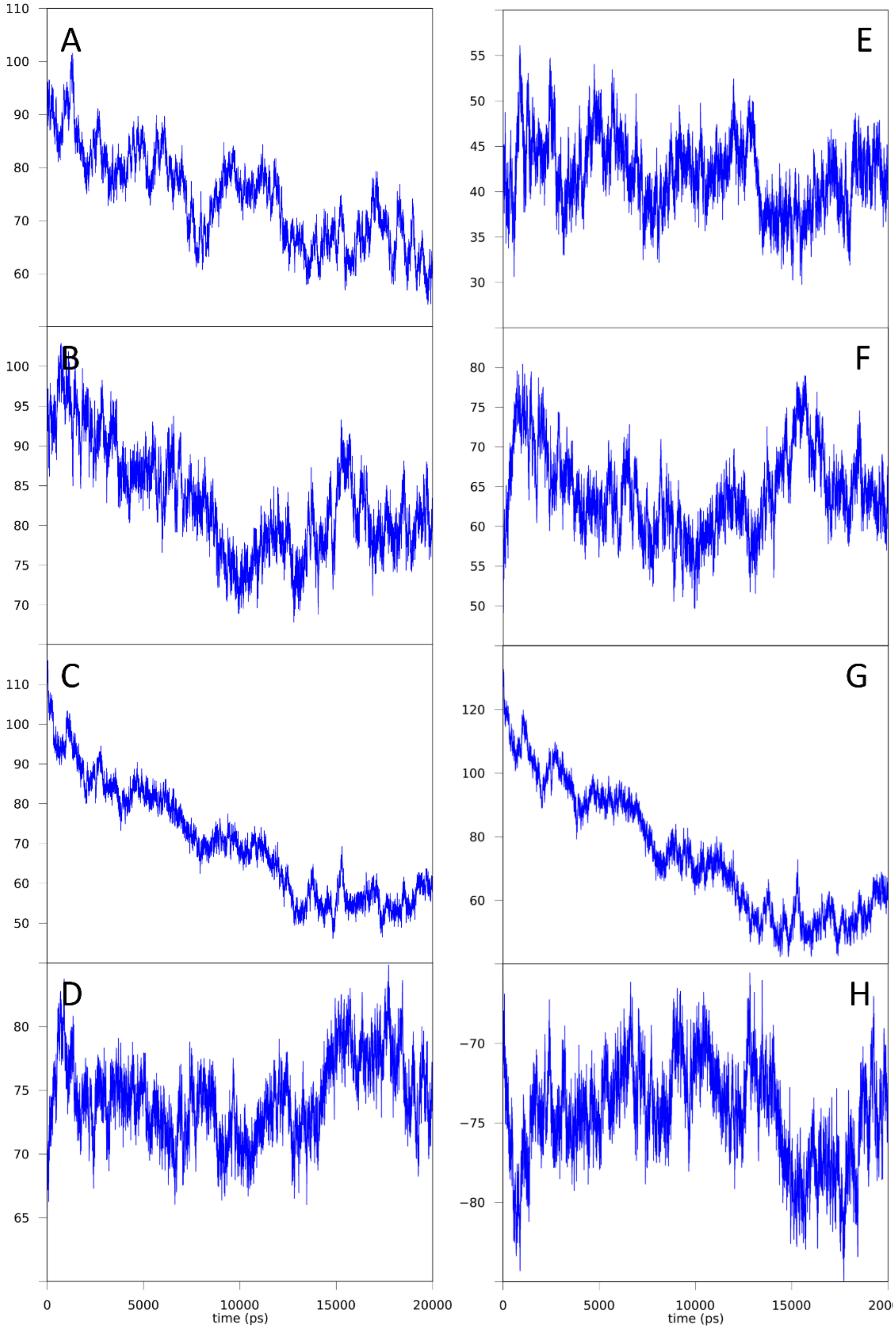

Figure S9. Relative orientation of individual domains as defined through the (dihedral) angles (in degree) in Table S4 as a function of time for simulation Q347E.

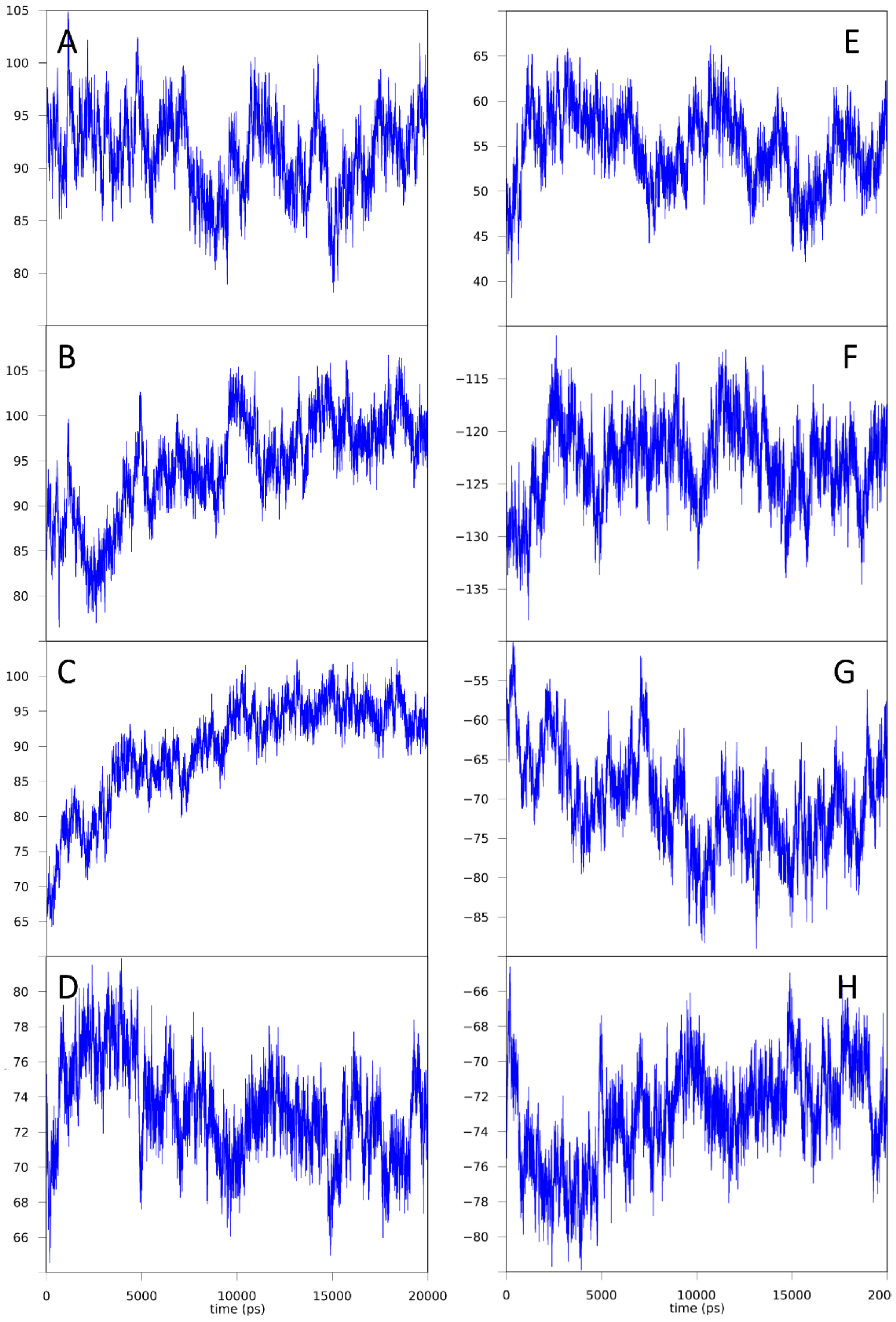

Figure S10. (**A**) Glycan tree structure that is attached to chain A of simulation B7 with atom numbering from topology used in simulation. Note, the glycan tree structure is connected through atom 620 and atom 2129. Simulation B4, H4, H7 and Q347E used topologies following a similar numbering scheme for the glycan tree structures. Actual atom numbering are shifted slightly depending on the simulation. (**B**) Glycan tree structure residue numbering according to the topology of simulation B4 and simulation B7. (**C**) Glycan tree structure residue numbering according to the topology of simulation H4, simulation H7 and simulation Q347E.

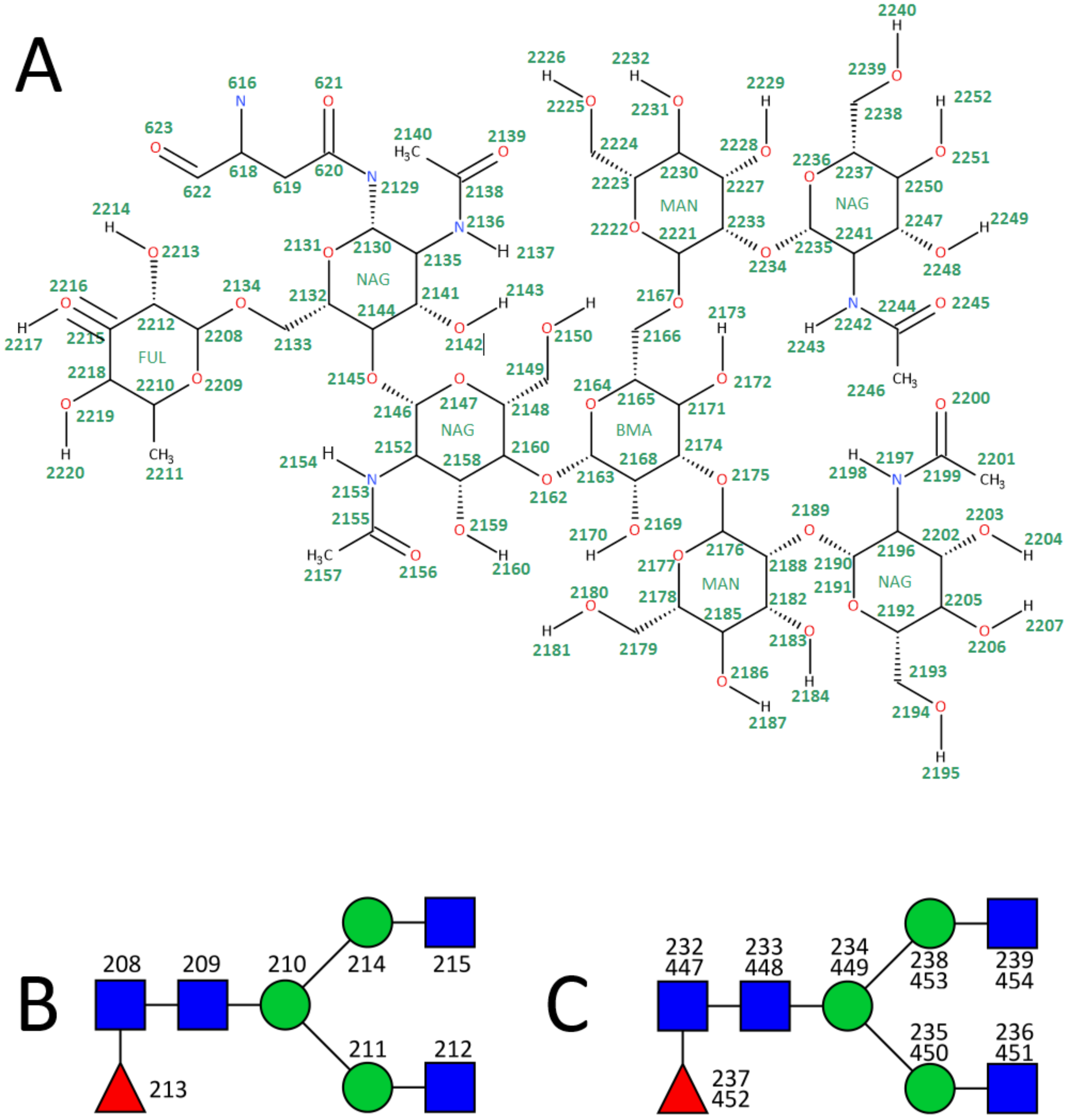

Figure S11. Schematic representation of an Fc fragment. Two CH2 domains (CH2A and CH2B), two CH3 domains (CH3A and CH3B) and two glycan structures (S1 and S2). Every domain or glycan, x, is represented by its position, $\vec{r}_x$, and its largest principle component, $\vec{n}_x$. The orientation of the domains can subsequently be described by a set of (dihedral) angles, as outlined in Table S4.

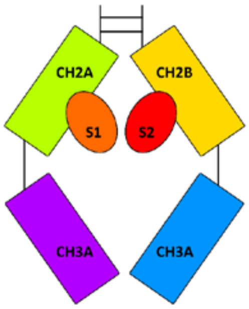

2. Building Blocks and Topologies

2.1. Atom Numbering Overview

Figure S12. Atom numbering for building blocks FUC, FUL, MAN and BMA.

α-L-fucose (FUC)/O1–O4

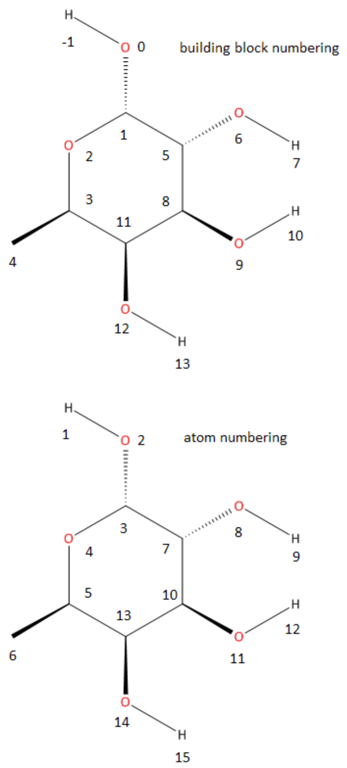

β-L-fucose (FUL)/O1–O4

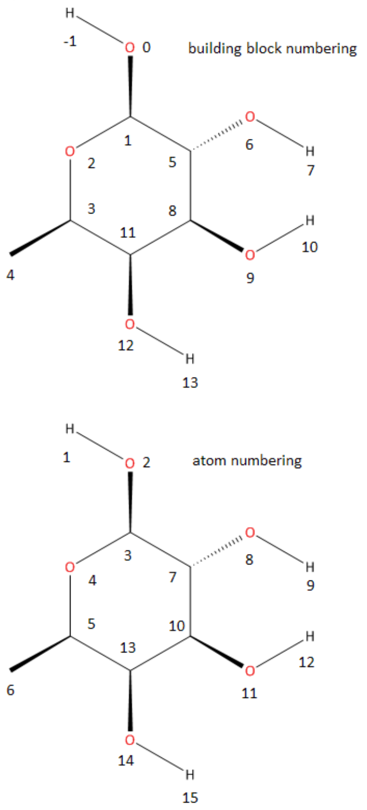

α-D-mannose (MAN)/O1–O2

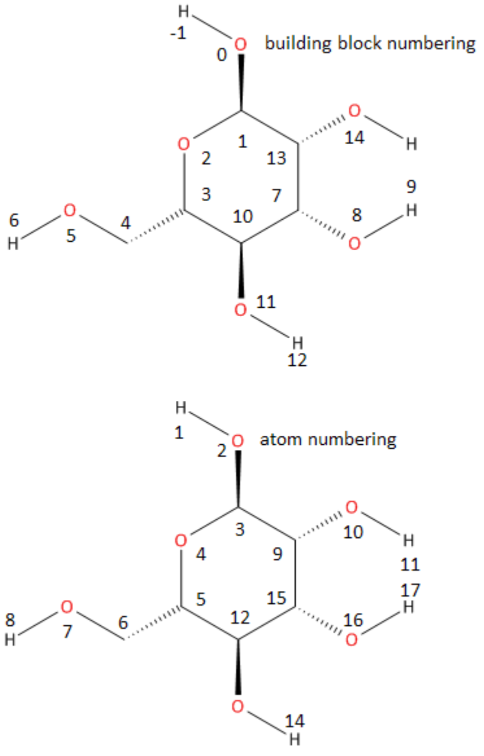

β-D-mannose (BMA)/O1–O3

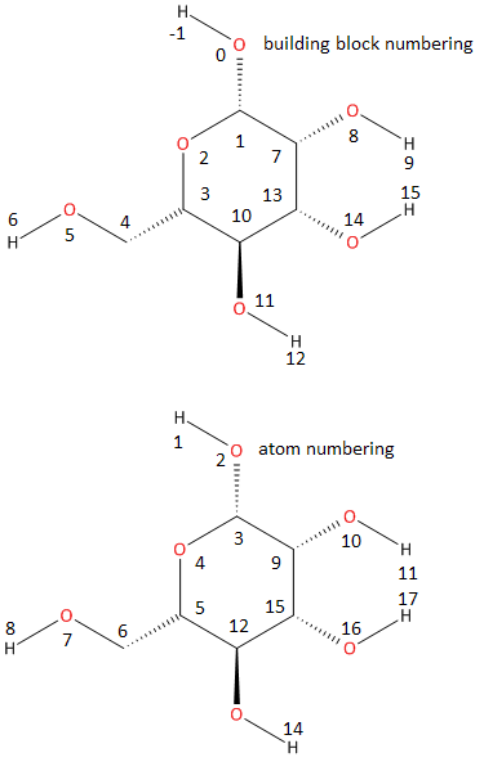

Figure S13. Atom numbering for building blocks NAG and NDG.

N-acetyl-D-glucosamine (NAG)/O1–O4

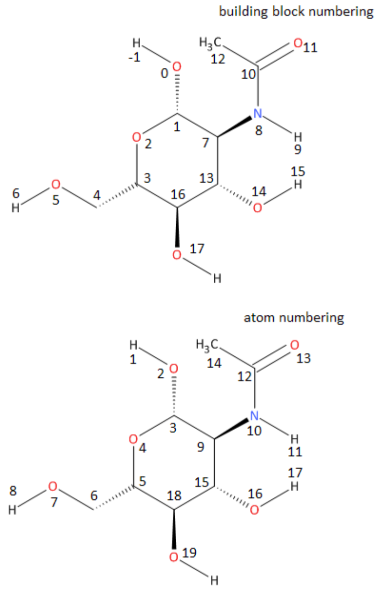

2-(acetylamino)-2-deoxy-A-D-glucopyranose (NDG)/O1–O4

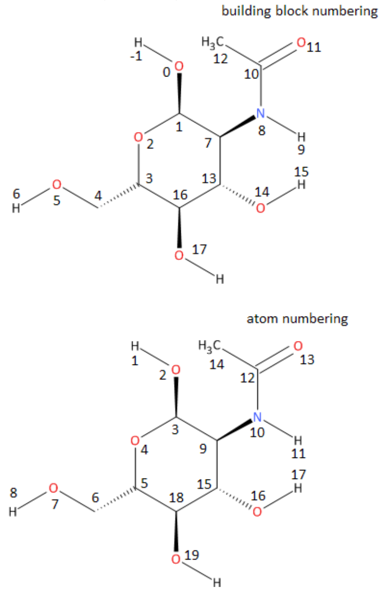

2.2. α-L-Fucose (FUC) Building Block

Figure S14. Graphical representation of building block FUC. Linkage is O1–O4.

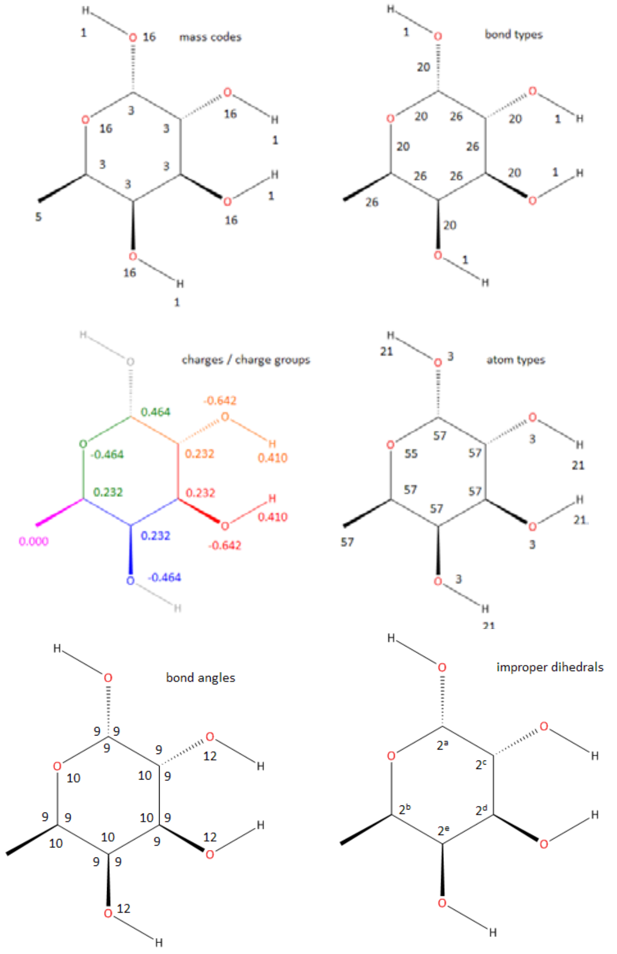

```
MTBUILDBLSOLUTE
# building block (residue, nucleotide, etc.)
# RNME
FUC
#(beta-L-fucose)
# number of atoms, number of preceding exclusions
# NMAT,NLIN
   12    2
# preceding exclusions
#ATOM                               MAE MSAE
   -1                                2 0 1
    0                                3 1 2 5
# atoms
#ATOM ANM  IACM MASS        CGMICGM MAE MSAE
    1 C1      57    3    0.46400   0   5    2    3    5    6    8
    2 O5      55   16   -0.46400   0   4    3    4    5   11
    3 C5      57    3    0.23200   1   4    4    8   11   12
    4 C6      57    5    0.00000   1   1   11
    5 C2      57    3    0.23200   0   5    6    7    8    9   11
    6 O2       3   16   -0.64200   0   2    7    8
    7 H2      21    1    0.41000   1   0
    8 C3      57    3    0.23200   0   4    9   10   11   12
    9 O3       3   16   -0.64200   0   2   10   11
   10 H3      21    1    0.41000   1   0
   11 C4      57    3    0.23200   0
   12 O4       3   16   -0.46400   0
# bonds
#  NB
   13
#  IB   JB  MCB
    0    1   20
    1    2   20
    1    5   26
    2    3   20
    3    4   26
    3   11   26
    5    6   20
    5    8   26
    6    7    1
    8    9   20
    8   11   26
    9   10    1
```

```
   11   12   20
# bond angles
# NBA
  19
#  IB   JB   KB  MCB
    0    1    2    9
    0    1    5    9
    2    1    5    9
    1    2    3   10
    2    3    4    9
    2    3   11    9
    4    3   11   10
    1    5    6    9
    1    5    8   10
    6    5    8    9
    5    6    7   12
    5    8    9    9
    5    8   11   10
    9    8   11    9
    8    9   10   12
    3   11    8   10
    3   11   12    9
    8   11   12    9
   11   12   13   12
# improper dihedrals
# NIDA
    5
#  IB   JB   KB   LB  MCB
    1    0    2    5    2
    3    2   11    4    2
    5    1    8    6    2
    8    5    9   11    2
   11    3    8   12    2
# dihedrals
# NDA
    17
#  IB   JB   KB   LB  MCB
 −1  0  1  2 46
 −1  0  1  2 48
  0  1  2  3 49
  5  1  2  3 46
  0  1  5  6 56
```

```
  0  1  5  8 34
  2  1  5  6 57
  1  2  3 11 46
  2  3 11  8 34
  2  3 11 12 57
  1  5  6  7 47
  1  5  8 11 34
  6  5  8  9 56
  5  8  9 10 47
  5  8 11  3 34
  9  8 11 12 56
  3 11 12 13 46
# ljexceptions
# NEXP
  19
# IJ JJ MCJ
 −1  2  1 1 1 21
 −1  8  2 1 1 21
 −1  3  2 1 1 21
  7 11  2 0
  2  7  2 0
  1 10  2 0
  3 10  2 0
  5 13  2 1 2 21
  2 13  2 1 2 21
  1  4  3 0
  4  8  3 0
  0  8  4 1 1 3
  0  3  4 1 1 3
  6 11  4 0
  1  9  4 0
  3  9  4 0
  5 12  4 0
  0  4  5 1 1 3
  4  9  5 0
END
```

Notes: FUC and FUL/O1-O4 share same end blocks.

2.3. *β*-L-Fucose (FUL) Building Block

Figure S15. Graphical representation of building block FUL.Linkage is O1–O4.

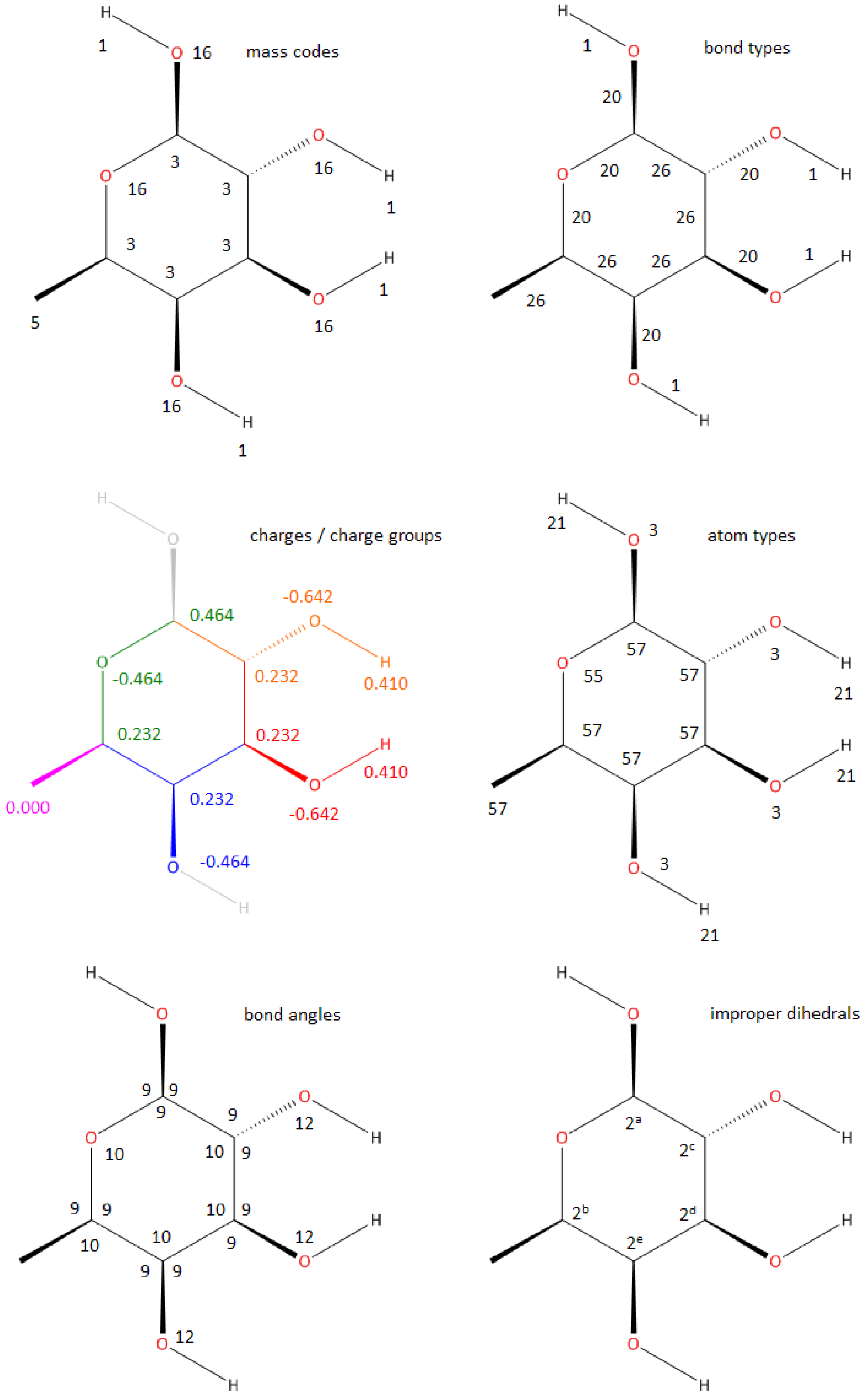

```
MTBUILDBLSOLUTE
# building block (residue, nucleotide, etc.)
# RNME
FUL
#(beta-L-fucose)
# number of atoms, number of preceding exclusions
# NMAT,NLIN
   12    2
# preceding exclusions
#ATOM                               MAE MSAE
   -1                                2 0 1
    0                                3 1 2 5
# atoms
#ATOM ANM  IACM MASS        CGMICGM MAE MSAE
    1 C1     57    3    0.46400   0   5    2    3    5    6    8
    2 O5     55   16   -0.46400   0   4    3    4    5   11
    3 C5     57    3    0.23200   1   4    4    8   11   12
    4 C6     57    5    0.00000   1   1   11
    5 C2     57    3    0.23200   0   5    6    7    8    9   11
    6 O2      3   16   -0.64200   0   2    7    8
    7 H2     21    1    0.41000   1   0
    8 C3     57    3    0.23200   0   4    9   10   11   12
    9 O3      3   16   -0.64200   0   2   10   11
   10 H3     21    1    0.41000   1   0
   11 C4     57    3    0.23200   0
   12 O4      3   16   -0.46400   0
# bonds
#  NB
   13
#  IB   JB  MCB
    0    1   20
    1    2   20
    1    5   26
    2    3   20
    3    4   26
    3   11   26
    5    6   20
    5    8   26
    6    7    1
    8    9   20
    8   11   26
    9   10    1
```

```
   11   12   20
# bond angles
# NBA
  19
#  IB   JB   KB  MCB
    0    1    2    9
    0    1    5    9
    2    1    5    9
    1    2    3   10
    2    3    4    9
    2    3   11    9
    4    3   11   10
    1    5    6    9
    1    5    8   10
    6    5    8    9
    5    6    7   12
    5    8    9    9
    5    8   11   10
    9    8   11    9
    8    9   10   12
    3   11    8   10
    3   11   12    9
    8   11   12    9
   11   12   13   12
# improper dihedrals
# NIDA
    5
#  IB   JB   KB   LB  MCB
    1    0    5    2    2
    3    2   11    4    2
    5    1    8    6    2
    8    5    9   11    2
   11    3    8   12    2
# dihedrals
# NDA
    17
#  IB   JB   KB   LB  MCB
 -1  0  1  2 46
 -1  0  1  2 48
  0  1  2  3 49
  5  1  2  3 46
  0  1  5  6 56
```

```
  0  1  5  8 34
  2  1  5  6 57
  1  2  3 11 46
  2  3 11  8 34
  2  3 11 12 57
  1  5  6  7 47
  1  5  8 11 34
  6  5  8  9 56
  5  8  9 10 47
  5  8 11  3 34
  9  8 11 12 56
  3 11 12 13 46
# ljexceptions
# NEXP
  19
# IJ JJ MCJ
 −1  2  1 1 1 21
 −1  8  2 1 1 21
 −1  3  2 1 1 21
  7 11  2 0
  2  7  2 0
  1 10  2 0
  3 10  2 0
  5 13  2 1 2 21
  2 13  2 1 2 21
  1  4  3 0
  4  8  3 0
  0  8  4 1 1 3
  0  3  4 1 1 3
  6 11  4 0
  1  9  4 0
  3  9  4 0
  5 12  4 0
  0  4  5 1 1 3
  4  9  5 0
END
MTBUILDBLEND
# building block (residue, nucleotide, etc.)
# RNME
FUL+
# number of atoms, number of atoms to be replaced
# NMAT, NREP
```

```
    3    1
# atoms
#ATOM ANM  IACM MASS        CGMICGM MAE MSAE
    1 H1     21    1    0.41000   0   2    2    3
    2 O1      3   16   -0.64200   0   3    3    4    7
# replacing atoms
    3 C1     57    3    0.46400   0
# bonds
#  NB
    2
#  IB   JB  MCB
    1    2    1
    2    3   20
# bond angles
# NBA
    1
#  IB   JB   KB  MCB
    1    2    3   12
# improper dihedrals
# NIDA
    0
#  IB   JB   KB   LB  MCB
# dihedrals
# NDA
    2
#  IB   JB   KB   LB  MCB
    1    2    3    4   47
    1    2    3    4   48
# ljexceptions
# NEXP
0
# IJ JJ MCJ
END
MTBUILDBLEND
# building block (residue, nucleotide, etc.)
# RNME
FUL-
# number of atoms, number of atoms to be replaced
# NMAT,NREP
    3   -2
# atoms
#ATOM ANM  IACM MASS        CGMICGM MAE MSAE
```

```
    1 C4      57    3    0.23200   0   2    2    3
    2 O4       3   16   -0.64200   0   1    3
    3 H4      21    1    0.41000   1   0
# bonds
#  NB
    2
#  IB   JB  MCB
    1    2   20
    2    3    1
# bond angles
# NBA
    1
#  IB   JB   KB  MCB
    1    2    3   12
# improper dihedrals
# NIDA
    0
#  IB   JB   KB   LB  MCB
# dihedrals
# NDA
    1
#  IB   JB   KB   LB  MCB
    0    1    2    3   47
# ljexceptions
# NEXP
0
# IJ JJ MCJ
END
```

2.4. β-D-Mannose (BMA) Building Block

Figure S16. Graphical representation of building block BMA. Linkage is O1–O3.

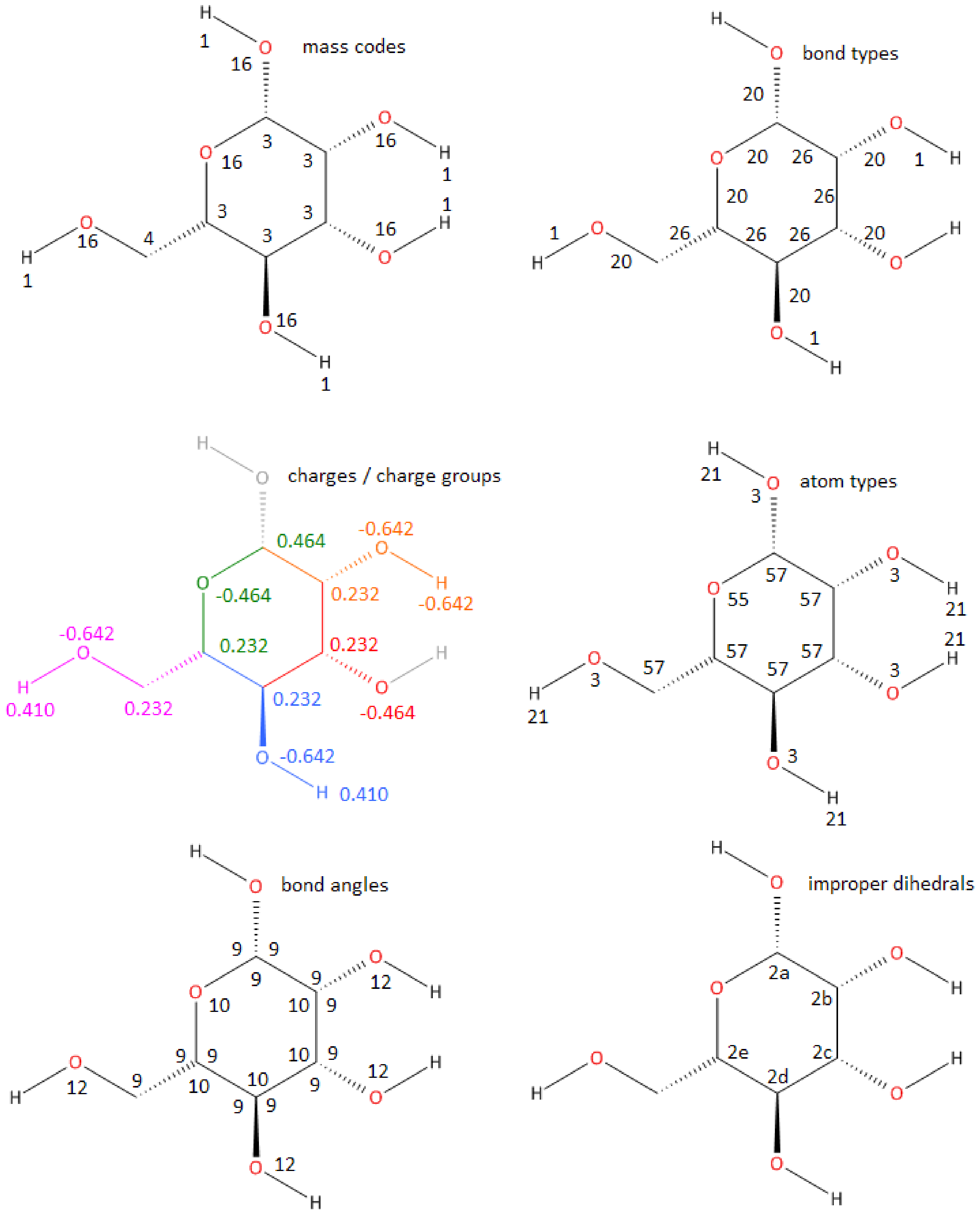

```
MTBUILDBLSOLUTE
# building block (residue, nucleotide, etc.)
# RNME
BMA
#(beta-D-mannose)
# number of atoms, number of preceding exclusions
# NMAT,NLIN
   14    2
# preceding exclusions
#ATOM                               MAE MSAE
   -1                                2 0 1
    0                                3 1 2 7
# atoms
#ATOM ANM  IACM MASS        CGMICGM MAE MSAE
    1 C1      57     3    0.46400   0   5    2    3    7    8   13
    2 O5      55    16   -0.46400   0   4    3    4    7   10
    3 C5      57     3    0.23200   1   5    4    5   10   11   13
    4 C6      57     4    0.23200   0   3    5    6   10
    5 O6       3    16   -0.64200   0   1    6
    6 H6      21     1    0.41000   1   0
    7 C2      57     3    0.23200   0   5    8    9   10   13   14
    8 O2       3    16   -0.64200   0   2    9   13
    9 H2      21     1    0.41000   1   0
   10 C4      57     3    0.23200   0   4   11   12   13   14
   11 O4       3    16   -0.64200   0   2   12   13
   12 H4      21     1    0.41000   1   0
   13 C3      57     3    0.23200   0
   14 O3       3    16   -0.46400   0
# bonds
#  NB
   15
#  IB   JB  MCB
    0    1   20
    1    2   20
    1    7   26
    2    3   20
    3    4   26
    3   10   26
    4    5   20
    5    6    1
    7    8   20
    7   13   26
```

```
     8    9    1
    10   11   20
    10   13   26
    11   12    1
    13   14   20
# bond angles
# NBA
    21
#  IB   JB   KB  MCB
     0    1    2    9
     0    1    7    9
     2    1    7    9
     1    2    3   10
     2    3    4    9
     2    3   10    9
     4    3   10   10
     3    4    5    9
     4    5    6   12
     1    7    8    9
     1    7   13   10
     8    7   13    9
     7    8    9   12
     3   10   11    9
     3   10   13   10
    11   10   13    9
    10   11   12   12
     7   13   10   10
     7   13   14    9
    10   13   14    9
    13   14   15   10
# improper dihedrals
# NIDA
     5
#  IB   JB   KB   LB  MCB
     1    0    2    7    2
     3    2    4   10    2
     7    1   13    8    2
    10    3   13   11    2
    13    7   10   14    2
# dihedrals
# NDA
    22
```

```
#  IB    JB    KB    LB   MCB
 −1  0  1  2  46
 −1  0  1  2  48
  0  1  2  3  49
  7  1  2  3  46
  0  1  7  8  56
  0  1  7 13  34
  2  1  7  8  57
  1  2  3 10  46
  2  3  4  5  56
  2  3  4  5  34
  2  3  4  5  55
 10  3  4  5  54
  2  3 10 13  34
  2  3 10 11  56
  3  4  5  6  47
  1  7  8  9  47
  1  7 13 10  34
  8  7 13 14  56
 13 10 11 12  47
  3 10 13  7  34
 11 10 13 14  56
  7 13 14 15  46
# ljexceptions
# NEXP
  19
# IJ JJ MCJ
 −1  2  1 1 1 21
 −1 13  2 1 1 21
 −1  3  2 1 1 21
  9 10  2 0
  2  9  2 0
  1 15  2 1 2 21
  3 15  2 1 2 21
  7 12  2 0
  2 12  2 0
  1  4  3 0
  4 13  3 0
  0 13  4 1 1 3
  0  3  4 1 1 3
  8 10  4 0
  1 14  4 0
```

```
  3 14  4 0
  7 11  4 0
  0  4  5 1 1 3
  4 14  5 0
END
MTBUILDBLEND
# building block (residue, nucleotide, etc.)
# RNME
BMA+
# number of atoms, number of atoms to be replaced
# NMAT,NREP
    3    1
# atoms
#ATOM ANM  IACM MASS        CGMICGM MAE MSAE
    1 H1     21    1    0.41000   0   2    2    3
    2 O1      3   16   -0.64200   0   3    3    4    9
# replacing atoms
    3 C1     57    3    0.46400   0
# bonds
#  NB
    2
#  IB   JB  MCB
    1    2    1
    2    3   20
# bond angles
# NBA
    1
#  IB   JB   KB  MCB
    1    2    3   12
# improper dihedrals
# NIDA
    0
#  IB   JB   KB   LB  MCB
# dihedrals
# NDA
    2
#  IB   JB   KB   LB  MCB
    1    2    3    4   47
    1    2    3    4   48
# ljexceptions
# NEXP
0
```

```
# IJ JJ MCJ
END
MTBUILDBLEND
# building block (residue, nucleotide, etc.)
# RNME
BMA-
# number of atoms, number of atoms to be replaced
# NMAT,NREP
    3   -2
# atoms
#ATOM ANM  IACM MASS        CGMICGM MAE MSAE
    1 C3     57    3    0.23200   0   2    2    3
    2 O3      3   16   -0.64200   0   1    3
    3 H3     21    1    0.41000   1   0
# bonds
#  NB
    2
#  IB   JB  MCB
    1    2   20
    2    3    1
# bond angles
# NBA
    1
#  IB   JB   KB  MCB
    1    2    3   12
# improper dihedrals
# NIDA
    0
#  IB   JB   KB   LB  MCB
# dihedrals
# NDA
    1
#  IB   JB   KB   LB  MCB
    0    1    2    3   47
# ljexceptions
# NEXP
0
# IJ JJ MCJ
END
```

2.5. α-D-Mannose(MAN) Building Block

Figure S17. Graphical representation of building block MAN. Linkage is O1–O2.

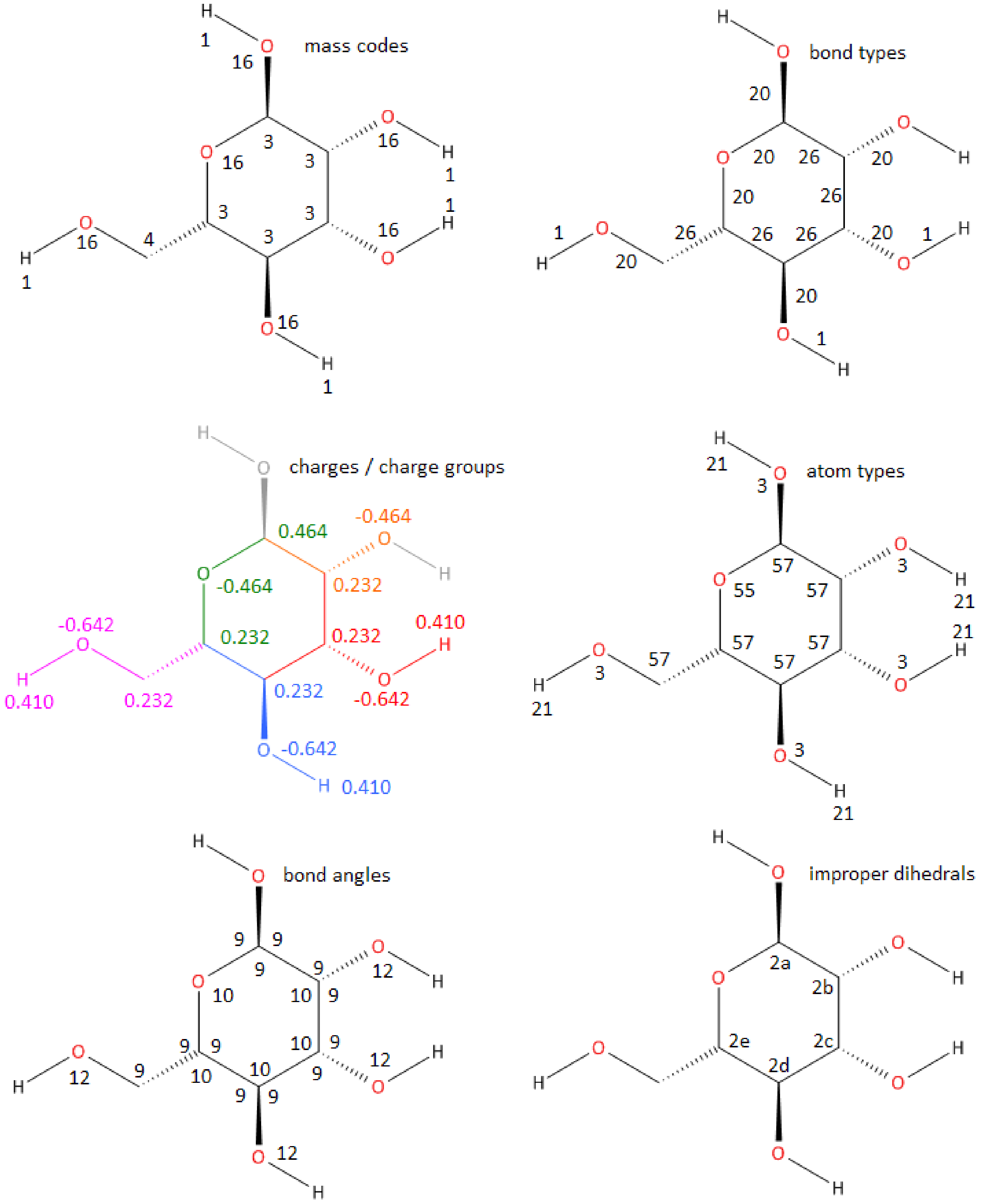

```
MTBUILDBLSOLUTE
# building block (residue, nucleotide, etc.)
# RNME
MAN
#(beta-D-mannose)
# number of atoms, number of preceding exclusions
# NMAT,NLIN
   14    2
# preceding exclusions
#ATOM                               MAE MSAE
   -1                                2 0 1
    0                                3 1 2 13
# atoms
#ATOM ANM  IACM MASS        CGMICGM MAE MSAE
    1 C1     57    3    0.46400   0   5    2    3    7   13   14
    2 O5     55   16   -0.46400   0   4    3    4   10   13
    3 C5     57    3    0.23200   1   5    4    5    7   10   11
    4 C6     57    4    0.23200   0   3    5    6   10
    5 O6      3   16   -0.64200   0   1    6
    6 H6     21    1    0.41000   1   0
    7 C3     57    3    0.23200   0   6    8    9   10   11   13
14
    8 O3      3   16   -0.64200   0   3    9   10   13
    9 H3     21    1    0.41000   1   0
   10 C4     57    3    0.23200   0   3   11   12   13
   11 O4      3   16   -0.64200   0   1   12
   12 H4     21    1    0.41000   1   0
   13 C2     57    3    0.23200   0
   14 O2      3   16   -0.46400   0
# bonds
#  NB
   15
#  IB   JB  MCB
    0    1   20
    1    2   20
    1   13   26
    2    3   20
    3    4   26
    3   10   26
    4    5   20
    5    6    1
    7    8   20
```

```
    7   13   26
    8    9    1
   10   11   20
   10    7   26
   11   12    1
   13   14   20
# bond angles
# NBA
   21
#  IB   JB   KB  MCB
    0    1    2    9
    0    1   13    9
    2    1   13    9
    1    2    3   10
    2    3    4    9
    2    3   10    9
    4    3   10   10
    3    4    5    9
    4    5    6   12
   10    7   13   10
    8    7   13    9
    8    7   10    9
    7    8    9   12
    3   10   11    9
    3   10    7   10
   11   10    7    9
   10   11   12   12
    1   13   14    9
    1   13    7   10
   14   13    7    9
   13   14   15   10
# improper dihedrals
# NIDA
    5
#  IB   JB   KB   LB  MCB
    1    0   13    2    2
    3    2    4   10    2
   13    1    7   14    2
   10    3    7   11    2
    7   13   10    8    2
# dihedrals
# NDA
```

```
   22
#  IB    JB    KB    LB  MCB
 −1  0  1  2  46
 −1  0  1  2  48
  0  1  2  3  49
 13  1  2  3  46
  0  1 13 14  56
  0  1 13  7  34
  2  1 13 14  57
  1  2  3 10  46
  2  3  4  5  56
  2  3  4  5  55
  2  3  4  5  34
 10  3  4  5  54
  2  3 10  7  34
  2  3 10 11  56
  3  4  5  6  47
 13  7  8  9  47
  1 13  7 10  34
  8  7 13 14  56
  7 10 11 12  47
  3 10  7 13  34
 11 10  7  8  56
  7 13 14 15  46
# ljexceptions
# NEXP
  19
# IJ JJ MCJ
 −1  2  1 1 1 21
 −1  7  2 1 1 21
 −1  3  2 1 1 21
 10 15  2 1 2 21
  2 15  2 1 2 21
  1  9  2 0
  3  9  2 0
 12 13  2 0
  2 12  2 0
  1  4  3 0
  4  7  3 0
  0  7  4 1 1 3
  0  3  4 1 1 3
 10 14  4 0
```

```
  1  8  4 0
  3  8  4 0
 11 13  4 0
  0  4  5 1 1 3
  4  8  5 0
END
MTBUILDBLEND
# building block (residue, nucleotide, etc.)
# RNME
MAN+
# number of atoms, number of atoms to be replaced
# NMAT,NREP
    3    1
# atoms
#ATOM ANM  IACM MASS        CGMICGM MAE MSAE
    1 H1     21    1    0.41000   0   2    2    3
    2 O1      3   16   -0.64200   0   3    3    4   15
# replacing atoms
    3 C1     57    3    0.46400   0
# bonds
#  NB
    2
#  IB   JB  MCB
    1    2    1
    2    3   20
# bond angles
# NBA
    1
#  IB   JB   KB  MCB
    1    2    3   12
# improper dihedrals
# NIDA
    0
#  IB   JB   KB   LB  MCB
# dihedrals
# NDA
    2
#  IB   JB   KB   LB  MCB
    1    2    3    4   47
    1    2    3    4   48
# ljexceptions
# NEXP
```

```
0
# IJ JJ MCJ
END
MTBUILDBLEND
# building block (residue, nucleotide, etc.)
# RNME
MAN-
# number of atoms, number of atoms to be replaced
# NMAT,NREP
    3   -2
# atoms
#ATOM ANM  IACM MASS        CGMICGM MAE MSAE
    1 C2     57    3    0.23200   0   2    2    3
    2 O2      3   16   -0.64200   0   1    3
    3 H2     21    1    0.41000   1   0
# bonds
#  NB
    2
#  IB   JB  MCB
    1    2   20
    2    3    1
# bond angles
# NBA
    1
#  IB   JB   KB  MCB
    1    2    3   12
# improper dihedrals
# NIDA
    0
#  IB   JB   KB   LB  MCB
# dihedrals
# NDA
    1
#  IB   JB   KB   LB  MCB
    0    1    2    3   47
# ljexceptions
# NEXP
0
# IJ JJ MCJ
END
```

2.6. N-*Acetyl*-D-*Glucosamine (NAG) Building Block*

Figure S18. Graphical representation of building block NAG. Linkage is O1–O4.

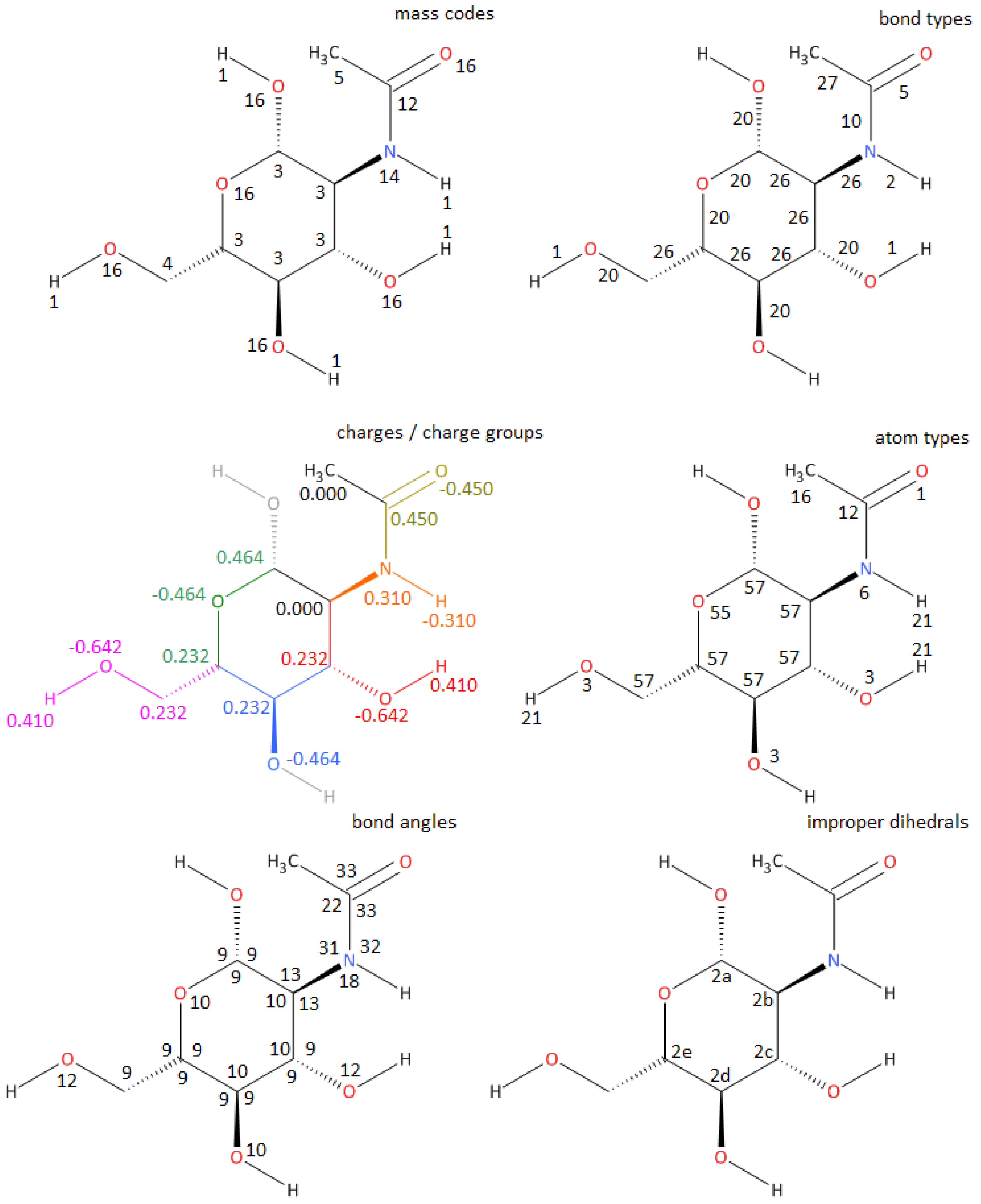

```
MTBUILDBLSOLUTE
# building block (residue, nucleotide, etc.)
# RNME
NAG
#(N-acetyl-D-glucosamine)
# number of atoms, number of preceding exclusions
# NMAT,NLIN
   17    2
# preceding exclusions
#ATOM                               MAE MSAE
   -1                                2 0 1
    0                                3 1 2 7
# preceding exclusions
#ATOM                               MAE MSAE
# atoms
#ATOM ANM  IACM MASS        CGMICGM MAE MSAE
    1 C1     57    3    0.46400   0   5    2    3    7    8   13
    2 O5     55   16   -0.46400   0   4    3    4    7   16
    3 C5     57    3    0.23200   1   5    4    5   13   16   17
    4 C6     57    4    0.23200   0   3    5    6   16
    5 O6      3   16   -0.64200   0   1    6
    6 H6     21    1    0.41000   1   0
    7 C2     57    3    0.00000   1   6    8    9   10   13   14
16
    8 N2      6   14   -0.31000   0   5    9   10   11   12   13
    9 H2     21    1    0.31000   1   1   10
   10 C7     12   12    0.45000   0   2   11   12
   11 O7      1   16   -0.45000   1   1   12
   12 C8     16    5    0.00000   1   0
   13 C3     57    3    0.23200   0   4   14   15   16   17
   14 O3      3   16   -0.64200   0   2   15   16
   15 H3     21    1    0.41000   1   0
   16 C4     57    3    0.23200   0
   17 O4      3   16   -0.46400   0
# bonds
#  NB
   18
#  IB   JB  MCB
    0    1   20
    1    2   20
    1    7   26
    2    3   20
```

```
     3    4   26
     3   16   26
     4    5   20
     5    6    1
     7    8   26
     7   13   26
     8    9    2
     8   10   10
    10   11    5
    10   12   27
    13   14   20
    13   16   26
    14   15    1
    16   17   20
# bond angles
# NBA
    26
#   IB   JB   KB  MCB
     0    1    2    9
     0    1    7    9
     2    1    7    9
     1    2    3   10
     2    3    4    9
     2    3   16    9
     4    3   16    9
     3    4    5    9
     4    5    6   12
     1    7    8   13
     1    7   13   10
     8    7   13   13
     7    8    9   18
     7    8   10   31
     9    8   10   32
     8   10   11   33
     8   10   12   22
    11   10   12   33
     7   13   14    9
     7   13   16   10
    14   13   16    9
    13   14   15   12
     3   16   13   10
     3   16   17    9
```

```
   13   16   17    9
   16   17   18   10
# improper dihedrals
# NIDA
    7
#  IB   JB   KB   LB  MCB
    1    0    2    7    2
    3    2    4   16    2
    7    1    8   13    2
    8    7    9   10    1
   10    8   11   12    1
   13    7   16   14    2
   16    3   13   17    2
# dihedrals
# NDA
   24
#  IB   JB   KB   LB  MCB
 −1  0  1  2  46
 −1  0  1  2  48
  2  1  7 13  34
  0  1  7  8  56
  2  1  7  8  57
  3  2  1  7  46
  3  2  1  0  49
 16  3  2  1  46
 16  3  4  5  54
  2  3  4  5  34
  2  3  4  5  55
  2  3  4  5  56
  2  3 16 13  34
  2  3 16 17  57
  3  4  5  6  47
  1  7  8 10  43
  1  7  8 10  44
  1  7 13 16  34
  8  7 13 14  56
  7  8 10 12  14
  7 13 14 15  47
  7 13 16  3  34
 14 13 16 17  56
  3 16 17 18  46
# ljexceptions
```

```
# NEXP
#11
  16
# IJ JJ MCJ
 -1   2  1 1 1 21
 -1  13  2 1 1 21
 -1   3  2 1 1 21
  1  15  2 0
  3  15  2 0
  7  18  2 1 2 21
  2  18  2 1 2 21
  1   4  3 0
  4  13  3 0
  0  13  4 1 1 3
  0   3  4 1 1 3
  1  14  4 0
  3  14  4 0
  7  17  4 0
  0   4  5 1 1 3
  4  14  5 0
END
MTBUILDBLEND
# building block (residue, nucleotide, etc.)
# RNME
NAG+
# number of atoms, number of atoms to be replaced
# NMAT,NREP
    3    1
# atoms
#ATOM ANM  IACM MASS        CGMICGM MAE MSAE
    1 H1     21    1    0.41000   0   2    2    3
    2 O1      3   16   -0.64200   0   3    3    4    9
# replacing atoms
    3 C1     57    3    0.46400   0
# bonds
#  NB
    2
#  IB   JB  MCB
    1    2    1
    2    3   20
# bond angles
# NBA
```

```
    1
#  IB   JB   KB  MCB
    1    2    3   12
# improper dihedrals
# NIDA
    0
#  IB   JB   KB   LB  MCB
# dihedrals
# NDA
    2
#  IB   JB   KB   LB  MCB
    1    2    3    4   47
    1    2    3    4   48
# ljexceptions
# NEXP
0
# IJ JJ MCJ
END
MTBUILDBLEND
# building block (residue, nucleotide, etc.)
# RNME
NAG-
# number of atoms, number of atoms to be replaced
# NMAT,NREP
    3   -2
# atoms
#ATOM ANM  IACM MASS        CGMICGM MAE MSAE
    1 C4     57    3    0.23200   0   2    2    3
    2 O4      3   16   -0.64200   0   1    3
    3 H4     21    1    0.41000   1   0
# bonds
#  NB
    2
#  IB   JB  MCB
    1    2   20
    2    3    1
# bond angles
# NBA
    1
#  IB   JB   KB  MCB
    1    2    3   12
# improper dihedrals
```

```
# NIDA
    0
#  IB   JB   KB   LB  MCB
# dihedrals
# NDA
    1
#  IB   JB   KB   LB  MCB
    0    1    2    3   47
# ljexceptions
# NEXP
0
# IJ JJ MCJ
END
```

2.7. 2-(Acetylamino)-2-Deoxy-A-D-Glucopyranose (NDG) Building Block

Figure S19. Graphical representation of building block NDG. Linkage is O1–O4.

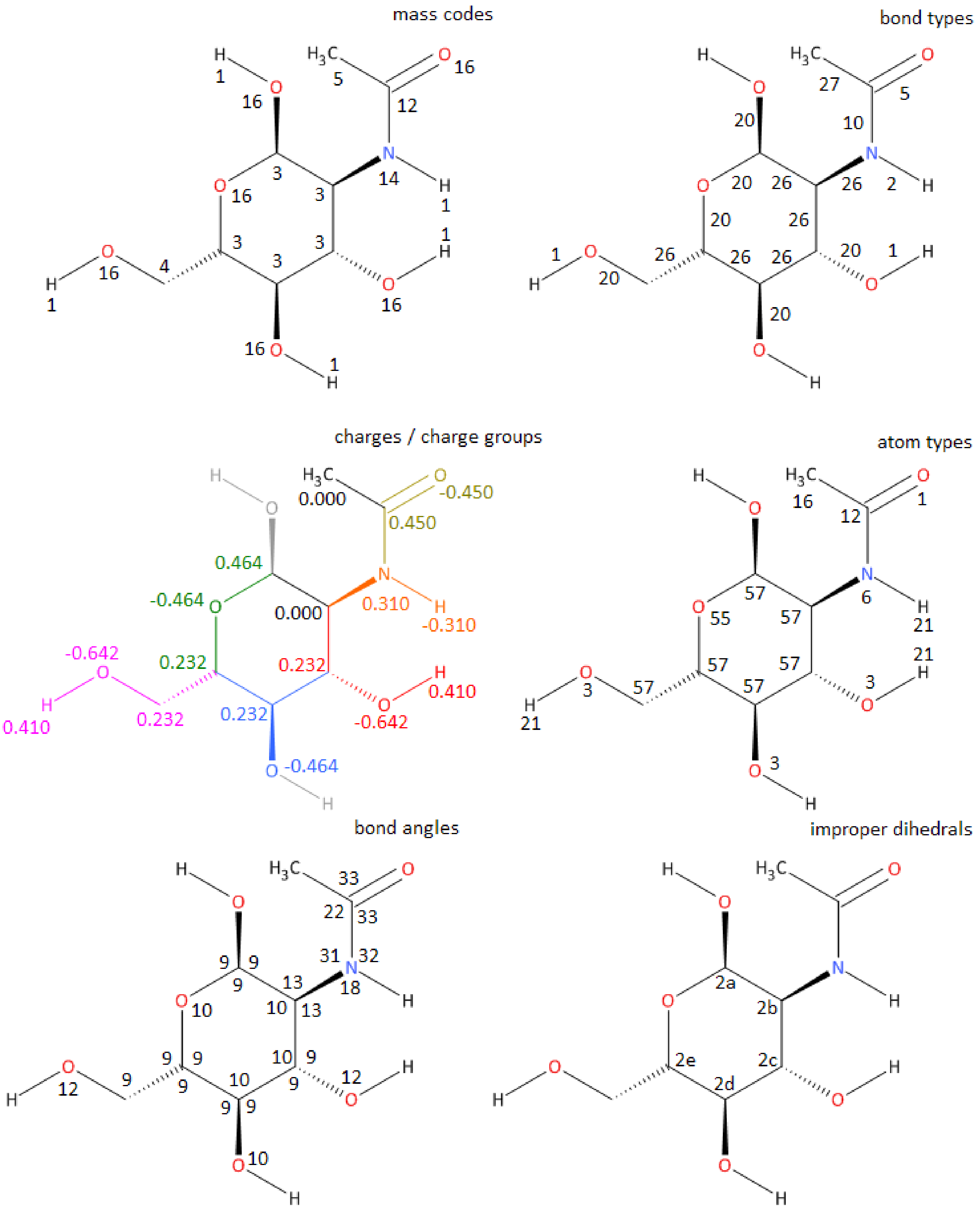

```
MTBUILDBLSOLUTE
# building block (residue, nucleotide, etc.)
# RNME
NDG
#(N-acetyl-D-glucosamine)
# number of atoms, number of preceding exclusions
# NMAT, NLIN
   17    2
# preceding exclusions
#ATOM                               MAE MSAE
   -1                                2 0 1
    0                                3 1 2 7
# preceding exclusions
#ATOM                               MAE MSAE
# atoms
#ATOM ANM  IACM MASS        CGMICGM MAE MSAE
    1 C1     57    3    0.46400   0    5    2    3    7    8   13
    2 O5     55   16   -0.46400   0    4    3    4    7   16
    3 C5     57    3    0.23200   1    5    4    5   13   16   17
    4 C6     57    4    0.23200   0    3    5    6   16
    5 O6      3   16   -0.64200   0    1    6
    6 H6     21    1    0.41000   1    0
    7 C2      57     3     0.00000    1    6     8     9    10    13    14
16
    8 N2      6   14   -0.31000   0    5    9   10   11   12   13
    9 H2     21    1    0.31000   1    1   10
   10 C7     12   12    0.45000   0    2   11   12
   11 O7      1   16   -0.45000   1    1   12
   12 C8     16    5    0.00000   1    0
   13 C3     57    3    0.23200   0    4   14   15   16   17
   14 O3      3   16   -0.64200   0    2   15   16
   15 H3     21    1    0.41000   1    0
   16 C4     57    3    0.23200   0
   17 O4      3   16   -0.46400   0
# bonds
#  NB
   18
#  IB   JB  MCB
    0    1   20
    1    2   20
    1    7   26
    2    3   20
```

```
     3    4   26
     3   16   26
     4    5   20
     5    6    1
     7    8   26
     7   13   26
     8    9    2
     8   10   10
    10   11    5
    10   12   27
    13   14   20
    13   16   26
    14   15    1
    16   17   20
# bond angles
# NBA
    26
#   IB   JB   KB  MCB
     0    1    2    9
     0    1    7    9
     2    1    7    9
     1    2    3   10
     2    3    4    9
     2    3   16    9
     4    3   16    9
     3    4    5    9
     4    5    6   12
     1    7    8   13
     1    7   13   10
     8    7   13   13
     7    8    9   18
     7    8   10   31
     9    8   10   32
     8   10   11   33
     8   10   12   22
    11   10   12   33
     7   13   14    9
     7   13   16   10
    14   13   16    9
    13   14   15   12
     3   16   13   10
     3   16   17    9
```

```
   13   16   17    9
   16 17 18 10
# improper dihedrals
# NIDA
    7
#  IB   JB   KB   LB  MCB
    1    0    7    2    2
    3    2    4   16    2
    7    1    8   13    2
    8    7    9   10    1
   10    8   11   12    1
   13    7   16   14    2
   16    3   13   17    2
# dihedrals
# NDA
   24
#  IB   JB   KB   LB  MCB
 −1  0  1  2  46
 −1  0  1  2  48
  2  1  7 13  34
  0  1  7  8  56
  2  1  7  8  57
  3  2  1  7  46
  3  2  1  0  49
 16  3  2  1  46
 16  3  4  5  54
  2  3  4  5  34
  2  3  4  5  55
  2  3  4  5  56
  2  3 16 13  34
  2  3 16 17  57
  3  4  5  6  47
  1  7  8 10  43
  1  7  8 10  44
  1  7 13 16  34
  8  7 13 14  56
  7  8 10 12  14
  7 13 14 15  47
  7 13 16  3  34
 14 13 16 17  56
  3 16 17 18  46
# ljexceptions
```

```
# NEXP
#11
  16
# IJ JJ MCJ
 −1   2  1 1 1 21
 −1  13  2 1 1 21
 −1   3  2 1 1 21
  1  15  2 0
  3  15  2 0
  7  18  2 1 2 21
  2  18  2 1 2 21
  1   4  3 0
  4  13  3 0
  0  13  4 1 1 3
  0   3  4 1 1 3
  1  14  4 0
  3  14  4 0
  7  17  4 0
  0   4  5 1 1 3
  4  14  5 0
END
```

© 2014 by the authors; licensee MDPI, Basel, Switzerland. This article is an open access article distributed under the terms and conditions of the Creative Commons Attribution license (http://creativecommons.org/licenses/by/3.0/).
